# Defining persistent critical illness based on growth trajectories in patients with sepsis

**DOI:** 10.1186/s13054-020-2768-z

**Published:** 2020-02-18

**Authors:** Zhongheng Zhang, Kwok M. Ho, Hongqiu Gu, Yucai Hong, Yunsong Yu

**Affiliations:** 1 Department of Emergency Medicine, Sir Run Run Shaw Hospital, Zhejiang University School of Medicine, Hangzhou, 310016 China; 2 Department of intensive care Medicine, Royal Perth Hospital, School of Population & Global Health, University of Western Australia, Crawley, Australia; 3 China National Clinical Research Center for Neurological Diseases; National Center for Healthcare Quality Management in Neurological Diseases, Beijing Tiantan Hospital, Capital Medical University, Beijing, 10070 China; 4 Department of Infectious Diseases, Sir Run Run Shaw Hospital, School of Medicine, Zhejiang University, Zhejiang, 310016 Hangzhou China; 5 Key Laboratory of Microbial Technology and Bioinformatics of Zhejiang Province, Hangzhou, Zhejiang, 310016 China

**Keywords:** Persistent critical illness, Sepsis, Unsupervised machine learning

## Abstract

**Background:**

Persistent critical illness is common in critically ill patients and is associated with vast medical resource use and poor clinical outcomes. This study aimed to define when patients with sepsis would be stabilized and transitioned to persistent critical illness, and whether such transition time varies between latent classes of patients.

**Methods:**

This was a retrospective cohort study involving sepsis patients in the eICU Collaborative Research Database. Persistent critical illness was defined at the time when acute physiological characteristics were no longer more predictive of in-hospital mortality (i.e., vital status at hospital discharge) than antecedent characteristics. Latent growth mixture modeling was used to identify distinct trajectory classes by using Sequential Organ Failure Assessment score measured during intensive care unit stay as the outcome, and persistent critical illness transition time was explored in each latent class.

**Results:**

The mortality was 16.7% (3828/22,868) in the study cohort. Acute physiological model was no longer more predictive of in-hospital mortality than antecedent characteristics at 15 days after intensive care unit admission in the overall population. Only a minority of the study subjects (*n* = 643, 2.8%) developed persistent critical illness, but they accounted for 19% (15,834/83,125) and 10% (19,975/198,833) of the total intensive care unit and hospital bed-days, respectively. Five latent classes were identified. Classes 1 and 2 showed increasing Sequential Organ Failure Assessment score over time and transition to persistent critical illness occurred at 16 and 27 days, respectively. The remaining classes showed a steady decline in Sequential Organ Failure Assessment scores and the transition to persistent critical illness occurred between 6 and 8 days. Elevated urea-to-creatinine ratio was a good biochemical signature of persistent critical illness.

**Conclusions:**

While persistent critical illness occurred in a minority of patients with sepsis, it consumed vast medical resources. The transition time differs substantially across latent classes, indicating that the allocation of medical resources should be tailored to different classes of patients.

**Electronic supplementary material:**

The online version of this article (10.1186/s13054-020-2768-z) contains supplementary material, which is available to authorized users.

## Introduction

Due to a variety of reasons such as hospital-acquired complications, endocrine dysregulation, unresolved inflammation, and protein catabolism, a substantial number of critically ill patients require prolonged intensive care unit (ICU) stay [1]. The term persistent critical illness (PCI) was coined referring to the situation when a patient’s initial critical condition was stabilized and resolved but the patient remained in the ICU due to a protracted recovery process. There is currently no standardized definition for PCI, but recently it has been suggested that it can be defined as the time at which acute physiological variables recorded at ICU arrival were no longer more predictive of mortality than antecedent characteristics [2, 3]. In the seminal paper by Iwashyna TJ and colleagues, substantial heterogeneity in the onset of PCI between different subgroups of a mixed ICU population—ranged from 7 to 22 days—was noted [2].

Sepsis is one of the leading causes of ICU admission, and through a number of mechanisms, it is also one of the most common causes of prolonged ICU stay. For instance, there is a large body of evidence showing that ICU-acquired infections occurred more frequently in patients with sepsis [4–6]. Sepsis is also a risk factor for ICU-acquired weakness and delirium [7–9]. Many of these hospital-acquired complications may contribute to a septic patient’s prolonged ICU stay [10–12], increasing the chance of developing PCI. Thus, the development of PCI in patients with sepsis is of special interests.

Although some authorities have suggested that PCI can be defined when a patient is still in ICU over a fixed time point such as 15 days [1], this arbitrary time point may vary between different septic patients due to sepsis case-mix heterogeneity by site of infection and more importantly by the number, type, and combinations of organ dysfunction [13]. Since PCI is associated with poor clinical outcome and the care of such patients are not cost-effective, alternative models of care (long term acute care hospitals), and payment reform have been considered to improve the care of these patients. Knowing which subgroup of patients will develop PCI can greatly improve the risk stratification and allocation of medical resources. More recently, biochemical signature of PCI was investigated and it showed that the changes in the urea-to-creatinine ratio could be a good biomarker for the development of PCI [14]. We hypothesized that the time point for transition from acute illness to PCI would vary significantly between different classes of septic patients, and this would depend heavily on the trajectories of the critical illness. In this retrospective multicenter cohort study, we aimed to define when patients admitted to an ICU with sepsis would be stabilized and transitioned to a state of persistent critical illness (PCI), and whether such transition time varied between subclasses of septic patients. The biochemical signature of PCI was also explored.

## Materials and methods

### Data source

The eICU Collaborative Research Database was used for the study. The database was a multi-center intensive care unit (ICU) database for over 200,000 admissions to 335 ICUs from 208 hospitals across the USA in 2014 and 2015 [15]. The database included data with high granularity, including vital sign measurements, care plan documentation, severity of illness measures, diagnosis information, treatment information, and laboratory variables. The database is released under the Health Insurance Portability and Accountability Act (HIPAA) safe harbor provision. The re-identification risk was certified as meeting safe harbor standards by Privacert (Cambridge, MA) (HIPAA Certification no. 1031219-2).

### Participants

Patients with a diagnosis of sepsis, recorded on the Acute Physiology and Chronic Health Evaluation (APACHE) IV dataset [16], on ICU admission were potentially eligible. In accordance with the Sepsis-3.0 criteria, sepsis was defined as suspected or documented infection plus an acute increase in SOFA score greater than 2 points [17, 18]. The cause of sepsis (i.e., site of infection) can be grouped into categories of gastrointestinal (GI), cutaneous/soft tissue, pulmonary, gynecologic, renal/UTI, unknown, and others.

### Variables

Variables recorded on the day of ICU entry were categorized into acute physiological variables and antecedent variables. The former included Glasgow coma score (GCS), bilirubin, creatinine, platelet, PaO_2_, FiO_2_, mean blood pressure, PaCO_2_, use of mechanical ventilation, urine output in 24 h, white blood cell count, temperature, respiratory rate, sodium, pH, heart rate, hematocrit, and plasma albumin, blood urea nitrogen (BUN), and glucose concentrations. The antecedent variables analyzed included age and sex. Comorbidities including acute immunodeficiency syndrome (AIDS), hepatic failure, lymphoma, metastatic cancer, leukemia, immunosuppression, and cirrhosis were extracted from the APACHE IV score. For some variables recorded more than once within the first 24 h after ICU admission, the one associated with the highest APACHE IV score (or acuity of illness) was employed. The Sequential Organ Failure Assessment (SOFA) score was computed using laboratory data recorded from day 1 to 10 after ICU admission. The SOFA score was used as the outcome variable in the latent growth mixture modeling in defining subgroups of septic patients with different trajectories after ICU admission.

### Management of missing data

Variables for calculating the SOFA score were recorded longitudinally and thus missing values were imputed by the incorporation of polynomials of time to fit a model to predict missing values. Intuitively, observed values close to the time of the missing value can greatly aid imputation of that value, whereas the data obtained not close to the timing of the missing data were given less weight in the imputation model [19]. For variables that were recorded on day 1 (cross-sectional variables), multiple imputations with the classification and regression trees (CART) method was employed [20, 21]. Variables with greater than 10% missing values were excluded from analysis (Additional file 1: Figure S1). Because many laboratory variables with missing values more than 10% (such as albumin, BUN and hematocrit) were presumed to be biochemical signature of PCI [14], they were included for sensitivity analysis. Variables including pH, PaCO_2_, and urine output were excluded from regression models.

### Statistical analysis

Outliers that could be regarded as an erroneous entry would be excluded from analysis (e.g., negative value of vital signs, age greater than 200, and urine output less than 0). Normally distributed continuous variables were expressed as mean and standard deviation (SD) and compared between groups using *t* test or analysis of variance. Skewed data were expressed as median and interquartile range (IQR) and were compared using non-parametric tests. Categorical data were expressed as the number and percentage and were compared between groups using the chi-square or Fisher exact test as appropriate.

Baseline variables recorded on ICU day 1 were categorized into two parts as described previously: acute and antecedent variables. Logistic regression models were developed separately for acute and antecedent variables, using mortality outcome as the response variable. The predictive performances of acute and antecedent models were evaluated from day 1 to day 28. A model evaluating the predictive performance of acute or antecedent variables after a certain day were fit on patients who had stayed in the hospital after that day. Thus, a total of 28 × 2 = 56 models were created. Each model was trained in 70% of the whole dataset, and then validated in the remaining 30% patients by reporting the area under the receiver operating characteristic curve (AUC). The splitting of the dataset into training and validation subsample was a random process and was performed for a number of iterations (by bootstrapping). Each iteration was different by having different subjects in the training and validating subsamples. The training-validation iteration was repeated for 100 times for each model, resulting in 100 AUC values for each model. The day on which PCI started was defined when the AUC values of the acute physiological variable models were not significantly greater than the antecedent variable models. Subjects who were still treated in ICU after the initiation of PCI were considered to have developed PCI, similar to what has been described in other studies [2, 3].

Latent growth mixture modeling assumes that the population is heterogeneous and composed of several latent classes of subjects characterized by a number of mean profiles of trajectories [22–25]. The best number of classes was determined by statistics such as Akaike information criterion (AIC), Bayesian information criteria (BIC), sample-adjusted BIC, and entropy. A smaller AIC, BIC SABIC, and entropy value indicated a better model fit [26]. Because a substantial number of patients are required for each class to be robust and clinically meaningful, 500 subjects was predefined as the minimum sample size required for each class (Additional file 1). The R package *lcmm* (version 1.7.9) was used for the latent growth mixture modeling.

Cox hazard model with time-dependent coefficient was employed to further test the hypothesis that the predictive performance of acute variables would attenuate with time. The conventional Cox proportional hazard model was extended by allowing the coefficient to vary over time [27, 28]. For the ease of interpretation, we specified a step function for *β*(*t*), i.e., different coefficients over different time intervals (0–48 h, 48–72 h, 72 h–7 days, 7–14 days, 14–21 days, and > 21 days). Two logistic regression models were built by regressing mortality on acute and antecedent variables, respectively. Acute variables were aggregated into an acute score reflecting the propensity to have the event conditional on these acute variables. Similarly, an antecedent score was calculated for each subject. Then both acute and antecedent scores were entered into the Cox hazard model with time-dependent coefficient (Additional file 1).

Clinical outcomes such as ICU and hospital length of stay (LOS) were compared across the latent classes. Other variables such as the day of developing PCI, percent of patients with PCI and discharge location were also compared between latent classes. All statistical analyses were performed using R (version 3.5.1). A two-tailed *p* value of less than 0.05 was considered to be statistically significant. The R code can be found at Additional file 2.

## Results

### Subjects and baseline characteristics

A total of 22,868 patients with sepsis were analyzed and a total of 3828 patients (16.7%) died before hospital discharge. The baseline characteristics between survivors and non-survivors are described in Table 1. While there was no significant difference on sex, ethnicity, height, and admission glucose, survivors were significantly younger (6 4± 18 vs. 70 ± 16 years; *p* <  0.001), were more likely to have renal/UTI infection (24.3% [4621/19,040] vs. 15.4% [591/3828]; *p* <  0.001), had a lower SOFA score (7 [6 to 9] vs. 10 [8 to 12]; *p* <  0.001), and were with less comorbidities than the non-survivors.

**Table 1:** baseline characteristics of hospital survivors and non-survivors

Variables	Survivors (*n* = 19,040)	Non-survivors (*n* = 3828)	*p*	SMD
Sex, male (%)	9653 (50.7%)	1947 (50.9%)	0.890	0.011
Age (years)	64 ± 18	70 ± 16	< 0.001	0.354
Ethnicity (%)			0.910	0.026
African American	1887 (9.9%)	395 (10.3%)		
Asian	365 (1.9%)	73 (1.9%)		
Caucasian	14,760 (77.5%)	2954 (77.2%)		
Hispanic	757 (4.0%)	163 (4.3%)		
Native American	170 (0.9%)	31 (0.8%)		
Other/unknown	1101 (5.8%)	212 (5.4%)		
Admission height (cm)	168.3 ± 14.3	167.8 ± 15.7	0.066	0.032
Admission weight (kg)	83.0 ± 28.5	79.0 ± 27.5	< 0.001	0.143
Source of infection (%)			< 0.001	0.276
GI	2264 (11.9%)	579 (15.1%)		
Cutaneous/soft tissue	1684 (8.8%)	219 (5.7%)		
Gynecologic	65 (0.3%)	10 (0.3%)		
Other	1192 (6.3%)	308 (8.0%)		
Pulmonary	7110 (37.3%)	1641 (42.9%)		
renal/UTI (including bladder)	4621 (24.3%)	591 (15.4%)		
unknown	2104 (11.1%)	480 (12.5%)		
Admitting source (%)			< 0.001	0.226
Operating room	55 (0.3%)	4 (0.1%)		
Recovery room	35 (0.2%)	4 (0.1%)		
Chest pain center	3 (0.0%)	0 (0.0%)		
Floor	3854 (20.2%)	1097 (28.7%)		
Other ICU	131 (0.7%)	40 (1.0%)		
Other hospital	377 (2.0%)	110 (2.9%)		
Direct admit	1115 (5.9%)	236 (6.2%)		
Emergency department	13,470 (70.7%)	2337 (61.1%)		
Unit type (%)			< 0.001	0.100
CCU-CTICU	1119 (5.9%)	220 (5.7%)		
CSICU	312 (1.6%)	67 (1.8%)		
CTICU	126 (0.7%)	36 (0.9%)		
Cardiac ICU	1094 (5.7%)	253 (6.6%)		
MICU	2475 (13.0%)	593 (15.5%)		
Med-Surg ICU	12,836 (67.4%)	2434 (63.6%)		
Neuro ICU	346 (1.8%)	87 (2.3%)		
SICU	732 (3.8%)	138 (3.6%)		
Use of mechanical ventilation (%)	3807 (20.0)	1641 (42.9)	< 0.001	0.508
GCS (median [IQR])	14 [10, 15]	10 [7, 14]	< 0.001	0.673
Bilirubin (mg/dl)	2.21 ± 2.46	2.85 ± 3.57	< 0.001	0.210
Creatinine (mg/dl)	1.89 ± 1.81	2.39 ± 1.72	< 0.001	0.285
Platelet (×10^9^/L)	197.15 ± 107.79	171.60 ± 113.49	< 0.001	0.231
PaO2 (mmHg)	93 ± 47	91 ± 53	0.132	0.025
Mean blood pressure (mmHg)	60 ± 16	50 ± 18	< 0.001	0.575
SOFA (median [IQR])	7 [6, 9]	10 [8, 12]	< 0.001	0.823
SOFA-respiratory (median [IQR])	2 [1, 3]	2 [2, 3]	< 0.001	0.420
SOFA-GSC (median [IQR])	1 [0, 2]	2 [1, 3]	< 0.001	0.668
SOFA-circulation (median [IQR])	2 [2, 2]	2 [2, 2]	< 0.001	0.159
SOFA-liver (median [IQR])	1 [0, 2]	1 [0, 2]	< 0.001	0.203
SOFA-coagulation (median [IQR])	0 [0, 1]	0 [0, 2]	< 0.001	0.351
SOFA-renal (median [IQR])	1 [0, 2]	2 [0, 2]	< 0.001	0.417
Dialysis (%)	947 (5.0%)	251 (6.6%)	< 0.001	0.068
Urine output (ml/24 h) (median [IQR])	1344 [642, 2380]	622 [176, 1232]	< 0.001	0.526
WBC (×10^9^/L) (median [IQR])	13.10 [8.3, 18.9]	14.80 [7.8, 22.2]	< 0.001	0.191
Temperature (°C) (median [IQR])	36.6 [36.2, 36.9]	36.4 [35.8, 36.8]	< 0.001	0.275
Respiratory rate (/min) (median [IQR])	31 [14, 38]	34 [27, 41]	< 0.001	0.233
Sodium (mmol/l)	137.99 ± 6.37	138.30 ± 7.58	0.007	0.045
Heart rate (/min) (median [IQR])	112 [98, 127]	120 [103, 137]	< 0.001	0.221
pH (mean ± SD)	7.36 ± 0.10	7.30 ± 0.14	< 0.001	0.537
Hematocrit (%)	31.0 ± 6.2	30.1 ± 7.0	< 0.001	0.149
Albumin (mg/dl)	2.53 ± 0.60	2.26 ± 0.62	< 0.001	0.448
PaCO2 (mmHg)	39.51 ± 12.69	39.71 ± 15.27	0.387	0.014
BUN (mg/dl)	33.24 ± 24.97	45.65 ± 28.71	< 0.001	0.461
Glucose (mg/dl)	162.80 ± 102.59	164.53 ± 111.84	0.349	0.016
AIDS (%)	53 (0.3%)	16 (0.4%)	0.202	0.024
Hepatic failure (%)	344 (1.8%)	150 (3.9%)	< 0.001	0.127
Lymphoma (%)	172 (0.9%)	53 (1.4%)	0.008	0.045
Metastatic cancer (%)	567 (3.0%)	233 (6.1%)	< 0.001	0.150
Leukemia (%)	255 (1.3%)	100 (2.6%)	< 0.001	0.092
Immunosuppression (%)	1004 (5.3%)	297 (7.8%)	< 0.001	0.101
Cirrhosis (%)	443 (2.3%)	207 (5.4%)	< 0.001	0.160

*Abbreviations*: *GI* gastrointestinal, *UTI* urinary tract infection, *ICU* intensive care unit, *CCU* coronary care unit, *CTICU* cardiothoracic intensive care unit, *CSICU* cardiac surgical intensive care, *MICU* medical ICU, *SICU* surgical ICU, *GCS* Glasgow coma scale, *SOFA* Sequential Organ Failure Assessment, *WBC* white blood cell count, *BUN* blood urea nitrogen, *AIDS* acquired immunodeficiency syndrome, *SD* standard deviation, *IQR* interquartile range, *SMD* standardized mean difference

### Latent growth mixture modeling

**Fig. 1:**
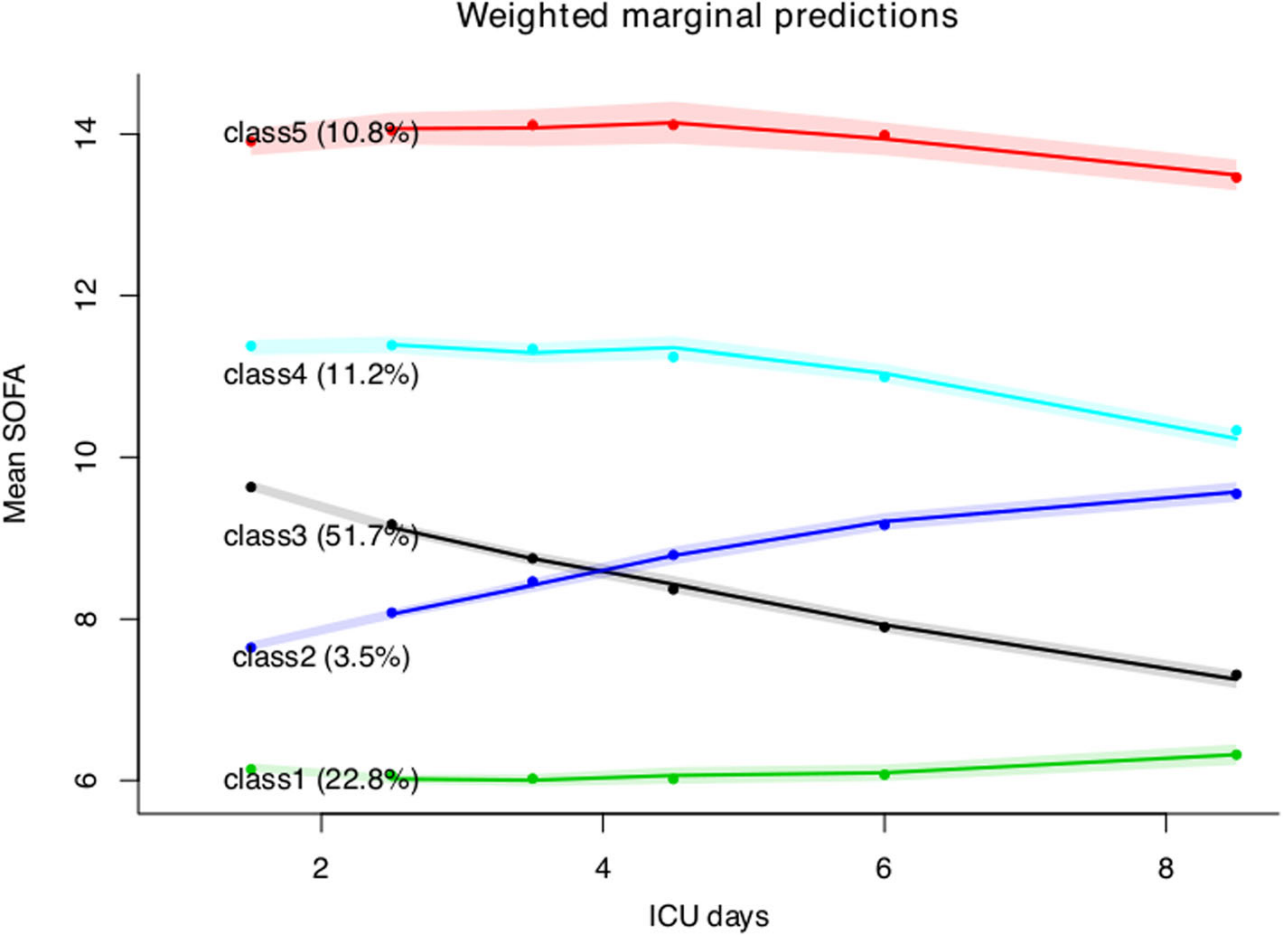
Five classes of sepsis identified by trajectories of SOFA score. The shaded area indicates the 95% confidence interval for each mean trajectory. The percentages in the parentheses indicate the percentages of patients each class accounts for. The number of classes was chosen based on model fit statistics. While classes 1 and 2 showed increasing SOFA score across ICU course, the remaining classes showed decreasing SOFA score. The initial SOFA scores (intercepts) were different among the five classes. Abbreviation: SOFA: Sequential Organ Failure Assessment

Model fit statistics are shown in Table 2. The AIC, BIC, and SABIC values declined continuously from a 1-class to a 6-class model, with the 5-class model having the lowest entropy. The 6-class model comprised a class with only 380 (1.66%) subjects and thus the 5-class model was considered the best fitted model. The trajectories of the 5 classes are shown in Fig. 1: class 1 (22.8%) was characterized by persistent low severity of illness, with a slightly increasing trend; class 2 (3.55%) was characterized by increasing severity of illness (or a lack of improvement to treatment with a very high mortality—70%); class 3 (51.7%) was characterized by moderate initial SOFA followed by decreasing severity of illness during the course of ICU stay; class 4 (11.2%) was characterized by high initial SOFA and slightly decreasing course; and class 5 (10.8%) was characterized by a persistent high severity of illness with a high mortality rate of 41.2%. Coefficients for the five quadratic functions are shown in Additional file 1: Table S1. The goodness-of-fit statistics for the 5-class model were maximum log-likelihood = − 189,328.67, AIC = 378,697.33, and BIC = 378,858.08.

**Table 2:** Statistics for choosing the best number of classes

Number of classes	Log likelihood	AIC	BIC	SABIC	Entropy	%class1	%class2	%class3	%class4	%class5	%class6
1	− 205,674.3	411,356.6	411,388.8	411,376.0	1.0000000	100.000000					
2	− 194,289.6	388,595.2	388,659.5	388,634.1	0.7755563	21.147455	78.852545				
3	−190,705.8	381,435.5	381,532.0	381,493.8	0.7120173	34.336190	58.649641	7.014168			
4	−189,735.3	379,502.6	379,631.2	379,580.4	0.6440044	3.961868	44.057198	12.663985	39.31695		
5	−189,328.7	378,697.3	378,858.1	378,794.5	0.5804752	22.787301	3.550813	51.670457	11.19468	10.796747	
6	− 188,823.6	377,695.3	377,888.2	377,811.9	0.5931242	34.445513	7.154102	41.770159	10.10145	4.871436	1.657338

*Abbreviations*: *AIC* Akaike information criterion, *BIC* Bayesian information criteria, *SABIC* sample-adjusted information criteria

### Persistent critical illness

**Fig. 2:**
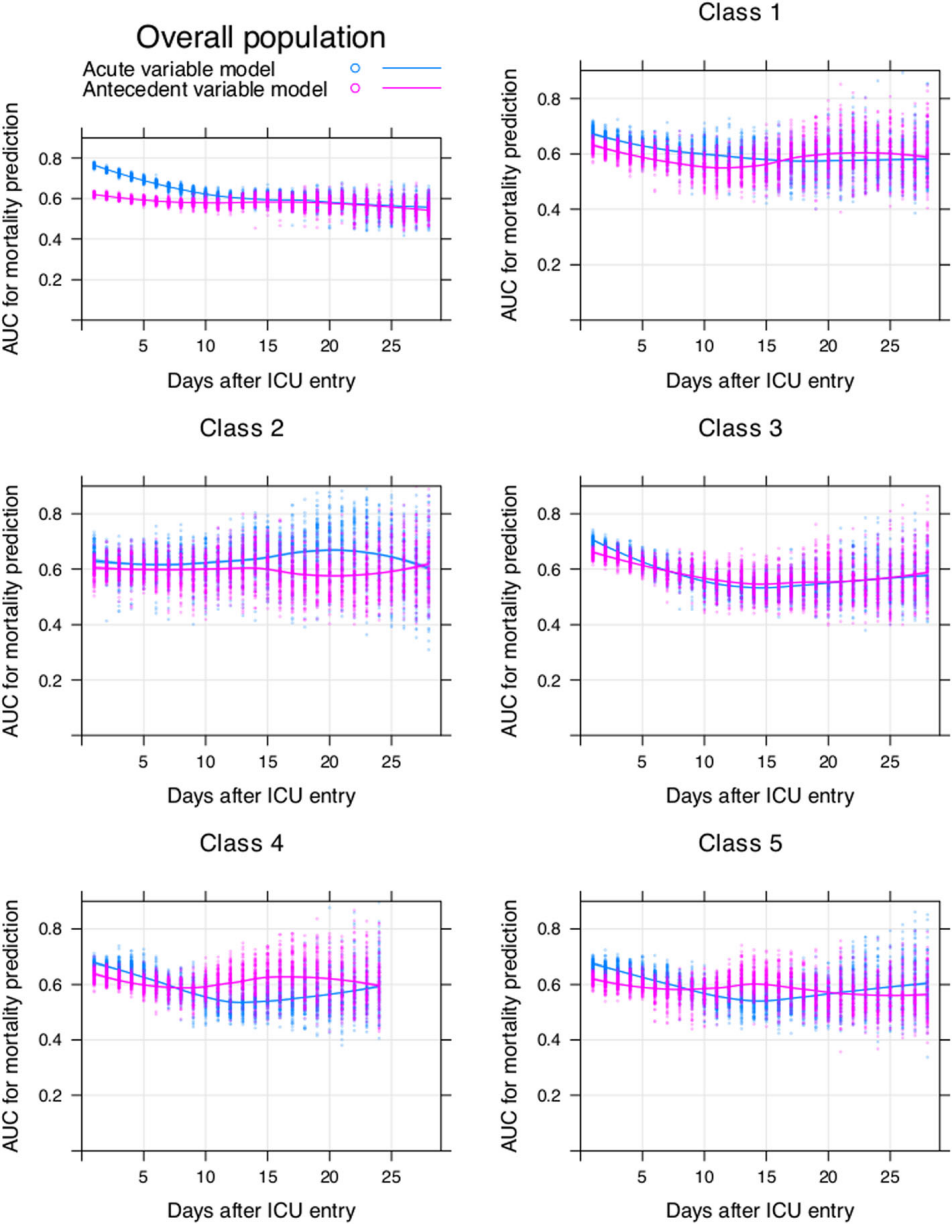
Comparisons of AUCs of acute and antecedent variable models in predicting hospital mortality. AUCs were calculated by splitting the whole sample into training (70%) and validating (30%) subsamples. Regression models were trained on the training sample and validated on the validating sample. The process iterated for 100 times for each model at each day, resulting in 2 × 100 = 200 circles at each day in the figure. The blue circles and lines represent the acute variable models, and the red ones represent the antecedent variable models

In the overall population, the discrimination of the acute physiological variable model was significantly better than the antecedent variable model between day 1 and day 15 (*p* <  0.001), but this was no longer true beyond day 15 (Fig. 2). Model discrimination was assessed in the testing dataset. The AUC was 0.764 (95% CI: 0.749 to 0.776) for the acute model versus 0.619 (95% CI 0.604 to 0.634) for the antecedent model on day 1. However, the AUCs were comparable for both models on day 21 (0.596 [95% CI 0.537 to 0.654] vs. 0.585 [95% CI 0.525 to 0.639], Additional file 1: Table S2). A total of 643 subjects (2.8%) developed PCI, accounting for 19% (15,834/83,125) and 10% (19,975/198,833) of the total ICU and hospital bed-days, respectively. Despite apparent stabilization with the development of PCI, the eventual hospital mortality rate of the those who developed PCI (163/643, 25%) was higher than those without PCI (3665/22,225, 16%) (*p* <  0.001).

For patients grouped in classes 1 and 2, transition to PCI occurred on days 16 and 27 after ICU admission (Table 3). Both classes 1 and 2 were characterized by increasing severity of illness in the ICU course, suggesting that the acute condition prompting ICU admission did not resolve precipitately with treatment. Classes 3, 4, and 5 were characterized by decreasing SOFA score and, thus, the transition to PCI started on days 6, 7, and 8, respectively which was earlier than those in classes 1 and 2 (e.g., septic response resolved with treatment quickly, and thus, the initial acute physiological characteristics were no longer predictive than antecedent characteristics within 10 days of ICU treatment). The percentage of patients developing PCI varied substantially across latent classes. While PCI was observed only in 1.9% and 3.6% of the patients in classes 1 and 2, respectively, over 20% of those in the classes 4 and 5 had PCI (Table 3). Baseline comparisons among the 5 latent classes showed that the antecedent variables were significantly different among the five classes (Additional file 1: Table S3). Class 2 showed great burden of comorbidities such as hepatic failure (9.4%), leukemia (4.7%) and cirrhosis (14.3%). Sensitivity analyses showed that the transition time for pulmonary infection and non-surgical patients were 13 and 20 days, respectively (Additional file 1: Figure S2 and S3).

**Table 3:** Differences of outcomes across the five latent classes

Clinical outcomes	Class 1 (*n* = 5211)	Class 2 (*n* = 812)	Class 3 (*n* = 11,816)	Class 4 (*n* = 2560)	Class 5 (*n* = 2469)	*p*
The day on which acute variables no longer more predictive than antecedent variables	16	27	6	7	8	
PCI, *n* (%)	97 (1.9%)	29 (3.6%)	831 (7.0%)	508 (19.8%)	584 (23.7%)	< 0.001
Hospital discharge location (%)						< 0.001
Unknown	6 (0.1%)	1 (0.1%)	17 (0.1%)	4 (0.2%)	3 (0.1%)	
Death	949 (18.2%)	568 (70.0%)	796 (6.7%)	497 (19.4%)	1018 (41.2%)	
Home	1986 (38.1%)	68 (8.4%)	6360 (53.8%)	897 (35.0%)	533 (21.6%)	
Nursing home	83 (1.6%)	8 (1.0%)	245 (2.1%)	52 (2.0%)	40 (1.6%)	
Other external	440 (8.4%)	46 (5.7%)	692 (5.9%)	211 (8.2%)	243 (9.8%)	
Other hospital	335 (6.4%)	41 (5.0%)	527 (4.5%)	159 (6.2%)	151 (6.1%)	
Rehabilitation	192 (3.7%)	27 (3.3%)	405 (3.4%)	112 (4.4%)	72 (2.9%)	
Skilled nursing facility	1220 (23.4%)	53 (6.5%)	2774 (23.5%)	628 (24.5%)	409 (16.6%)	
Unit discharge status, expired (%)	603 (11.6%)	485 (59.7%)	332 (2.8%)	277 (10.8%)	719 (29.1%)	
Length of stay in ICU (days) (median [IQR])	2 [1, 5]	4 [1, 10]	2 [1, 3]	3 [2, 6]	4 [1, 8]	< 0.001
Hospital discharge status, expired (%)	949 (18.2%)	568 (70.0%)	796 (6.7%)	497 (19.4%)	1018 (41.2%)	< 0.001
Length of stay in hospital (days) (median [IQR])	7 [3, 12]	5 [2, 14]	6 [4, 9]	7 [4, 12]	7 [3, 14]	< 0.001

*Abbreviations*: *PCI* persistent critical illness, *ICU* intensive care unit, *IQR* interquartile range

Note that the transition time for each trajectory class was different from that when the transition time was defined in the overall population (15 days). Such difference in transition time lead to the observation that the patients who were defined to have PCI in the overall population may not be defined to have PCI in the trajectory classes, and vice versa

The Cox hazard regression model with time-dependent coefficient showed that while the hazard ratio of acute score (i.e., the model for estimating acute score is shown in Additional file 1: Table S5) in predicting survival outcome was greater than the antecedent score (i.e., the model for estimating acute score is shown in Additional file 1: Table S6) over the initial 7 days (HR [95% CI] 1.60 [1.55, 1.65] vs. 1.29 [1.16, 1.44] for the initial 2 days; 2.10 [1.95, 2.26] vs. 1.73 [1.41, 2.12] for day 3; and 2.21 [2.10, 2.33] vs. 1.93 [1.67, 2.24] for days 3 to 7), the impact of acute score attenuated over time and was not better than the antecedent score after 14 days (1.37 [1.23, 1.53] vs. 2.38 [1.82, 3.13] for days 14 to 21, and 1.26 [1.12, 1.42] vs. 2.32 [1.77, 3.04] for over 21 days; Table 4).

**Table 4:** Baseline acute and antecedent variables in predicting survival outcome in a Cox regression model with time-dependent coefficient

Step function of time	Acute score		Antecedent score	
	HR (95% CI)	*p*	HR (95% CI)	*p*
0–48 h	1.60 [1.55, 1.65]	< 0.001	1.29 [1.16, 1.44]	< 0.001
48–72 h	2.10 [1.95, 2.26]	< 0.001	1.73 [1.41, 2.12]	< 0.001
72 h–7 days	2.21 [2.10, 2.33]	< 0.001	1.93 [1.67, 2.24]	< 0.001
7 days–14 days	1.54 [1.45, 1.64]	< 0.001	1.72 [1.45, 2.03]	< 0.001
14–21 days	1.37 [1.23, 1.53]	< 0.001	2.38 [1.82, 3.13]	< 0.001
> 21 days	1.26 [1.12, 1.42]	< 0.001	2.32 [1.77, 3.04]	< 0.001

The survival model was built with time dependent coefficients. The acute and antecedent variables were used to construct a predicting score by using logistic regression model

*Abbreviations*: *HR* hazard ratio, *CI* confidence interval

### Biochemical signature of PCI

**Fig. 3:**
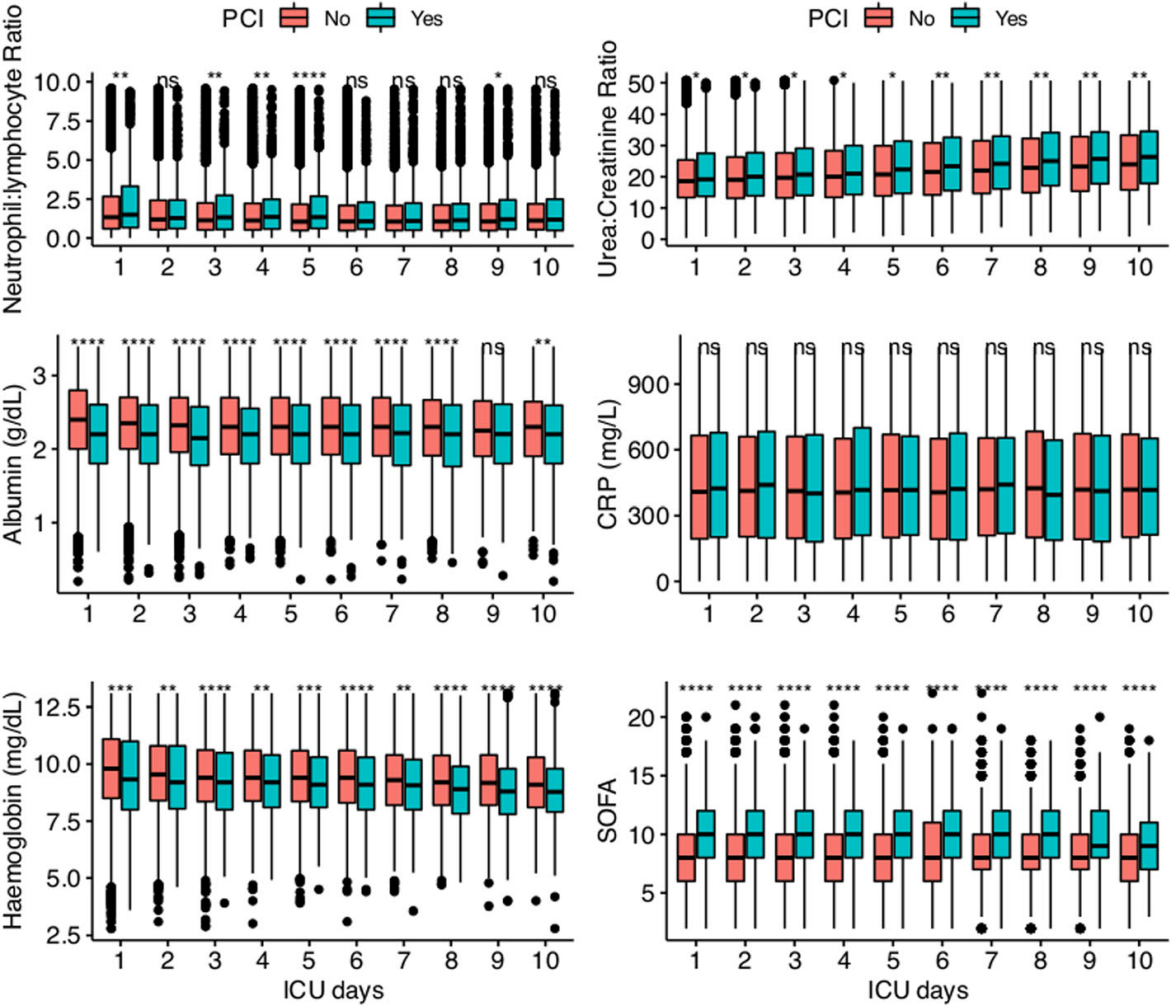
Biochemical signature of PCI versus non-PCI. The result showed that CRP was not significantly different between PCI versus non-PCI patients. Biochemical values of albumin and hemoglobin were consistently lower in the PCI group, whereas SOFA and urea-to-creatinine ratio were greater in the PCI group. More importantly, the magnitude of difference in urea-to-creatinine ratio appeared to increase from day 1 to 10 *< 0.05; **< 0.01; ***< 0.001; ****< 0.0001

**Fig. 4:**
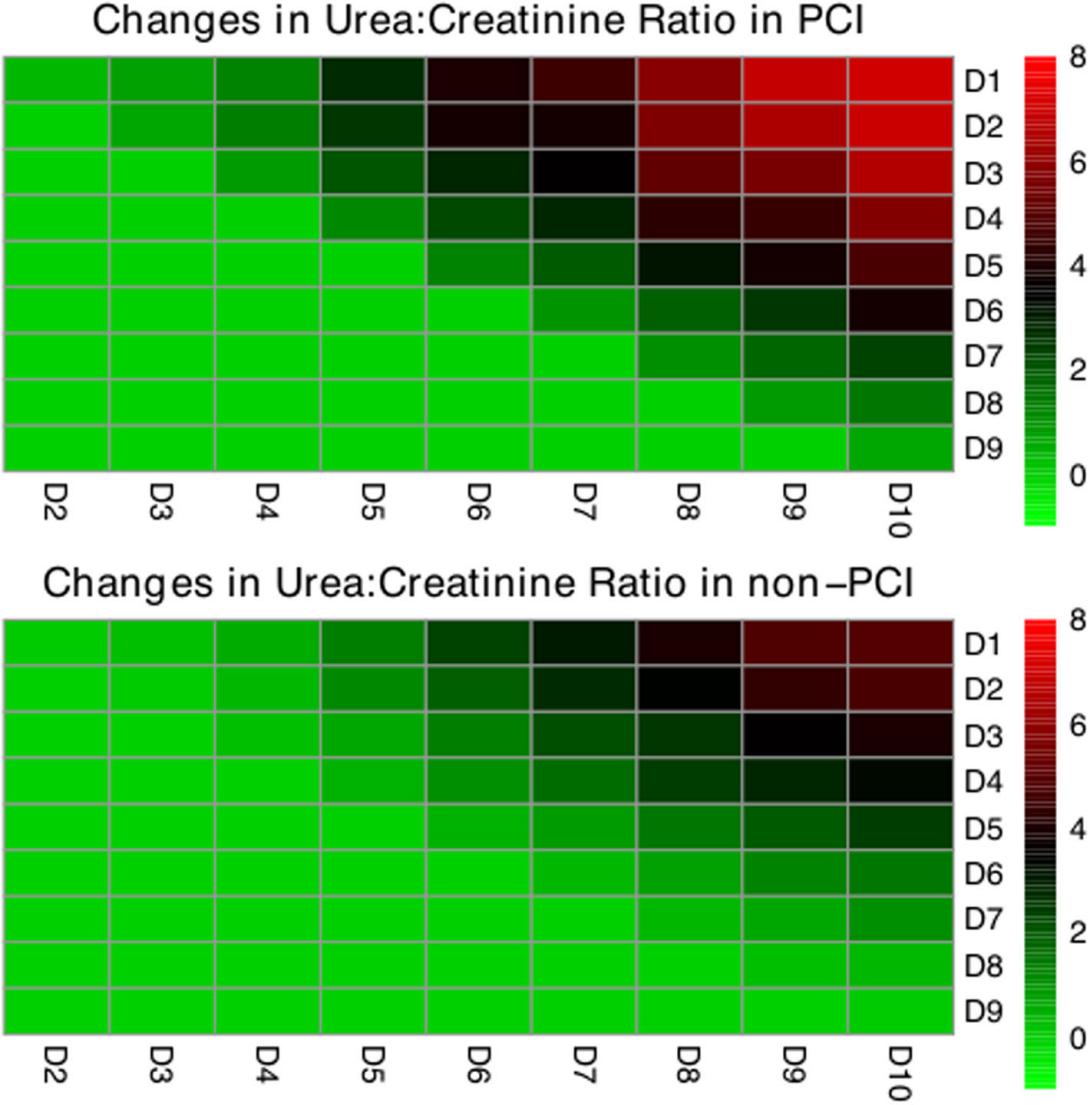
Heatmap showing the median changes in urea-to-creatinine ratio between different combinations of days. The row days represent the reference days, to which the column days were compared. Lighter red indicates greater magnitude of increases in urea-to-creatinine ratio. Cells below the diagonal is set to zero (green) because comparisons were only performed by values measured at later days minus early days

Patients with and without PCI were compared for the difference in biochemistry. It appeared that patients with PCI had significantly greater SOFA score than those without PCI over the first 10 days. Albumin and hemoglobin were significantly lower in the PCI group versus non-PCI group. The neutrophil-to-lymphocyte ratio was significantly greater in PCI group on days 1, 3, 4, 5, and 9. C-reactive protein (CRP) was not significantly different across all 10 days (Fig. 3). The changes in the urea-to-creatinine ratio were significantly greater in the PCI group than in the non-PCI group (Fig. 4). For example, patients with PCI showed significantly greater increase in the urea-to-creatinine ratio for day 4 (1.28 [− 4.03, 8.55] vs. 0.58 [− 5.08, 7.21]; *p* = 0.018) to day 10 (7.07 [− 1.34, 18.16] vs. 5 [− 3.52, 16.06]; *p* = 0.003) as compared to day 1 (Table 5). The same trend of changes in urea-to-creatinine ratio was observed in other time combinations. Patients with PCI were more likely to be discharged to other hospital (14% vs. 5%; *p* <  0.001) or rehabilitation centers (10% vs. 3%; *p* <  0.001) than the non-PCI group (Additional file 1: Table S6).

**Table 5:** Comparisons of changes in urea-to-creatinine ratio in PCI versus non-PCI patients

Changes in urea-to-creatinine ratio, median (IQR)	Total (*n* = 17,945)	Non-PCI (*n* = 17,302)	PCI (*n* = 643)	*p*
Day 1 as reference				
Day 2	0.1 (− 3.2, 3.49)	0.09 (− 3.22, 3.5)	0.37 (− 2.69, 3.27)	0.287
Day 3	0.3 (− 4.43, 5.48)	0.27 (− 4.45, 5.48)	0.75 (− 3.73, 5.33)	0.325
Day 4	0.64 (− 5, 7.28)	0.58 (− 5.08, 7.21)	1.28 (− 4.03, 8.55)	0.018
Day 5	1.49 (− 4.78, 9.41)	1.35 (− 4.92, 9.21)	2.8 (− 3.93, 11.27)	0.006
Day 6	2.54 (− 4.29, 11.37)	2.35 (− 4.55, 11.16)	3.99 (− 2.76, 12.64)	0.008
Day 7	3.33 (− 3.74, 12.67)	3.1 (− 4.07, 12.44)	4.58 (− 2.52, 14.04)	0.022
Day 8	4.31 (− 3.19, 14.55)	3.91 (− 3.58, 14.08)	5.9 (− 1.63, 15.77)	0.005
Day 9	5.33 (− 2.95, 16.24)	4.85 (− 3.59, 16.16)	6.87 (− 1.93, 16.53)	0.063
Day 10	5.85 (− 2.89, 16.75)	5 (− 3.52, 16.06)	7.07 (− 1.34, 18.16)	0.003
Day 2 as reference				
Day 3	0.11 (− 2.96, 3.17)	0.07 (− 3.03, 3.15)	0.69 (− 2.06, 3.39)	0.007
Day 4	0.44 (− 3.99, 5.41)	0.31 (− 4.14, 5.32)	1.4 (− 2.38, 6.47)	< 0.001
Day 5	1.27 (− 4.19, 7.97)	1.13 (− 4.38, 7.71)	2.56 (−2.31, 10)	< 0.001
Day 6	2.14 (− 4.01, 9.96)	1.85 (− 4.36, 9.59)	3.86 (− 2.54, 11.58)	< 0.001
Day 7	2.98 (− 3.61, 11.39)	2.73 (− 3.84, 11.07)	3.9 (−2.2, 13.16)	0.001
Day 8	3.98 (− 3.14, 13.21)	3.44 (− 3.79, 12.5)	5.64 (− 1.03, 14.39)	< 0.001
Day 9	4.99 (− 3.21, 15.09)	4.4 (−4.02, 14.93)	6.41 (−1.14, 15.82)	0.007
Day 10	5.45 (− 2.7, 16)	4.77 (−3.62, 15.4)	6.95 (−0.55, 17.46)	< 0.001
Day 3 as reference				
Day 4	0.27 (− 2.64, 3.2)	0.21 (− 2.71, 3.1)	0.85 (−1.74, 4.07)	< 0.001
Day 5	0.84 (− 3.26, 5.83)	0.67 (− 3.45, 5.58)	2 (− 1.83, 7.64)	< 0.001
Day 6	1.62 (− 3.57, 8.23)	1.39 (− 3.85, 7.81)	2.81 (−1.81, 9.98)	< 0.001
Day 7	2.41 (− 3.58, 10.23)	2.14 (− 4, 9.86)	3.52 (−1.76, 11.91)	< 0.001
Day 8	3.14 (− 3.19, 11.67)	2.58 (− 3.61, 11.06)	5.17 (−1.13, 13.55)	< 0.001
Day 9	4.23 (− 3.18, 13.65)	3.56 (− 4, 13.23)	5.53 (−1.1, 14.96)	0.001
Day 10	4.76 (− 2.92, 14.61)	3.91 (− 3.72, 13.65)	6.51 (−1.19, 16.23)	< 0.001
Day 4 as reference				
Day 5	0.55 (− 2.23, 3.64)	0.46 (− 2.36, 3.57)	1.12 (−1.37, 3.88)	< 0.001
Day 6	1.25 (− 2.96, 6.24)	1.05 (− 3.21, 6.04)	2.27 (−1.52, 7.27)	< 0.001
Day 7	1.95 (− 3.11, 8.29)	1.67 (− 3.42, 8.04)	2.86 (−1.97, 9.15)	0.004
Day 8	2.83 (− 2.96, 10.18)	2.44 (− 3.39, 9.8)	4.26 (−1.55, 11.39)	< 0.001
Day 9	3.36 (− 3, 11.9)	2.87 (−3.54, 11.57)	4.44 (−1.54, 13.06)	0.007
Day 10	4.19 (− 3.16, 13.54)	3.38 (−3.82, 12.77)	5.71 (−1.72, 14.76)	< 0.001
Day 5 as reference				
Day 6	0.59 (− 2.11, 3.71)	0.47 (− 2.24, 3.63)	1.22 (− 1.36, 3.98)	< 0.001
Day 7	1.06 (− 2.87, 5.96)	0.87 (− 3.13, 5.74)	2 (− 1.82, 6.69)	0.005
Day 8	1.88 (− 3.25, 8.04)	1.43 (− 3.58, 7.75)	3.11 (− 1.32, 8.47)	< 0.001
Day 9	2.6 (− 3.66, 9.87)	1.93 (− 4.02, 9.57)	3.88 (− 2.27, 10.42)	0.008
Day 10	3.15 (− 3.78, 11.18)	2.4 (− 4.32, 10.27)	4.71 (− 2.95, 12.71)	< 0.001
Day 6 as reference				
Day 7	0.46 (− 2.26, 3.56)	0.38 (−2.38, 3.46)	0.92 (− 1.54, 4.02)	0.011
Day 8	1.04 (− 2.83, 5.68)	0.7 (− 3.14, 5.41)	1.88 (− 1.65, 6.61)	< 0.001
Day 9	1.75 (− 3.7, 8.13)	1.21 (− 4.2, 7.71)	2.52 (− 2.03, 8.77)	0.002
Day 10	2.29 (− 4, 9.36)	1.46 (− 4.81, 8.64)	3.84 (− 2.28, 10.72)	< 0.001
Day 7 as reference				
Day 8	0.51 (− 2.2, 3.51)	0.32 (− 2.45, 3.33)	1.05 (− 1.46, 3.98)	< 0.001
Day 9	1 (− 3.26, 5.82)	0.69 (− 3.61, 5.7)	1.72 (− 2.26, 6.07)	0.009
Day 10	1.35 (− 3.48, 7.14)	1.04 (− 4.14, 6.67)	2.37 (− 2.32, 8.14)	< 0.001
Day 8 as reference				
Day 9	0.44 (− 2.45, 3.57)	0.23 (−2.65, 3.56)	0.82 (− 1.99, 3.75)	0.051
Day 10	0.66 (− 3.47, 5)	0.31 (− 3.94, 4.73)	1.49 (− 2.79, 5.38)	< 0.001
Day 9 vs 10	0.23 (− 2.53, 3.1)	0.02 (− 3.08, 2.98)	0.63 (− 1.73, 3.43)	< 0.001

*Abbreviations*: *IQR* interquartile range, *PCI* persistent critical illness

Note: Patients stayed in ICU for less than 2 days were excluded (e.g., no comparison could be performed)

## Discussion

The study empirically investigated the onset time of PCI in patients with sepsis. In the overall population, PCI started on average at 15 days after ICU admission. While there were only 2.8% subjects developed PCI, they accounted for 19% and 10% of the total ICU and hospital bed-days, respectively. The results of our study provide evidence to support our hypothesis that the onset of PCI varied substantially across different subgroups of septic patients reflecting substantial variations in the trajectory of sepsis. These results have some clinical implications and require further discussion. First, those with a declining trend in their severity of illness after ICU admission developed PCI at an earlier stage than those with increasing severity of illness despite ICU treatment. This finding is not surprising and confirms that a septic patient’s response to their initial ICU treatment has a bearing on when they can be stabilized to develop PCI.

Second, in a population-based study involving a mixed ICU population, Iwashyna TJ and colleagues found that the acute physiological characteristics obtained on day 1 progressively lost their mortality predictive power and were no longer better than the antecedent characteristics beyond day 10. Subgroup analysis in this study found that the transition to PCI for septic patients occurred earlier (7 vs 10 days) than the overall mixed ICU population. Our results showed that there was also significant heterogeneity between septic patients in their transition to PCI. Although those grouped in the latent classes 4 and 5 had developed transition to PCI at approximately 7 days similar to what was reported by Iwashyna et al., some septic patients—similar to those in latent classes 1 and 2—certainly would need much longer time before they can be stabilized and transitioned to PCI. Since Iwashyna’s study did not report the trajectory pattern of their sepsis patients, the difference cannot be fully explained. In another study [3], the results of subgroup analysis showed that surgical patients, especially those with cardiac surgery had significantly later transition time (20 days) to PCI—similar to our septic patients in the latent classes 1 and 2 in this study.

Conventionally, the definition of PCI was based on a fixed time point such as 14 days, after ICU entry [29], without considering the causes of prolonged ICU stay. When PCI is defined by the relative discriminative ability of the acute and antecedent characteristics, the reasons for the prolonged ICU stay are considered. If the cause of the prolonged ICU stay is mainly related to the primary reason for ICU admission, the discrimination of initial acute physiological variables will remain more important than the antecedent variables. Such patients would not be considered to have PCI even if they stay in ICU for more than 15 days.

In a web-based survey [30], most respondents believed that PCI should be defined as “those whose reason for being in the ICU was now more related to their ongoing critical illness than their original reason for admission to the ICU,” rather than by a fixed time point. Thus, it is reasonable to empirically define PCI as when the acute physiological characteristics are no longer more predictive than the antecedent characteristics. Our study has provided some evidence to show that the transition time to PCI varied substantially even within a homogenous diagnostic group such as sepsis, and trajectories of clinical course—as defined by the daily SOFA scores—explain their heterogeneity. It can be deduced that patients with decreasing SOFA score were those whose septic condition had stabilized or resolved, and the major reasons for an ongoing ICU stay related to their PCI are likely due to conditions such as delirium, ICU-acquired weakness and respiratory insufficiency that are not fully captured by daily SOFA score. For patients with a progressive increase in daily SOFA score, the primary septic process has not resolved and thus the acute physiological characteristics will remain predictive of mortality longer than in those who have responded to ICU treatment. Another evidence supporting the current approach to define PCI comes from epidemiological data that multiple organ failure syndrome (MOFS) has evolved into bimodal phenomenon with decreasing early and increasing late mortality [31–34].

Finally, we would like to acknowledge the limitations of the present study. Our database did not contain the data on the reasons for prolonged stay in ICU in our patients, even though delirium, ICU-acquired weakness, and respiratory insufficiency leading to slow weaning of mechanical ventilation are the most likely explanations. The relative importance of each of these complications in contributing to a prolonged ICU stay and PCI remains uncertain and deserves further investigation by an adequately powered prospective study. In addition, this study was not able to distinguish between secondary or ICU-acquired infection and unresolved primary infection. Because only patients in the USA were included in this study, it is uncertain whether our results are applicable to low- or middle-income countries where the practice of critical care is different. Our growth mixture model only included the first 10 days after ICU entry, and the temporal trend after 10 days was not known. However, we believe that it is reasonable to do so based on both theoretical and practical issues: (1) the latent trajectory classes need to be defined before PCI, and according to previous literature [2], the transition day generally occurs after 10 days; (2) we attempted to characterize the trajectory of critical illness in acute phase, and 10 days can capture this phase without extending to the chronic illness phase; (3) SOFA score is the outcome variable in the growth curve modeling and it reflects the sequential organ failure due to infection in acute phase; and (4) we need adequate sample size for the growth curve analysis (e.g., SOFA scores were not available for most patients after 10 days). Finally, the mortality was not considered in the growth mixture modeling since it could not be measured longitudinally over time. Instead, we used SOFA score as the outcome because it is a continuous variable and its mean trajectory can be modeled. Furthermore, the outcome variable SOFA can capture some aspects of the mortality because they are closely related to each other [35].

## Conclusions

In conclusion, the study found that a transition to PCI occurred, on average, on day 15 after ICU admission in patients with sepsis. This transition time varied substantially between latent classes primarily related to their course of critical illness or response to ICU treatment. Subjects showing a progressive decline in daily SOFA score had an earlier transition to PCI than those with increasing SOFA score; but the substantial variability between septic individuals we observed in this study suggested that accurate prediction of the onset of PCI in patients with sepsis is difficult. More research is needed to identify the best way or biomarkers to predict the onset of PCI.

## Supplementary information

Additional file 1: **Table S1.** Fixed effects in the longitudinal 5-class model. **Table S2.** Predictive performance of acute and antecedent models as represented by AUCs for the day 1, 2, 7, 14 and 21. **Table S3.** The differences of baseline variables across the 5 classes. **Table S4.** Binary logistic regression model using antecedent variables to predict mortality. **Table S5.** Binary logistic regression model using acute variables to predict mortality. **Table S6.** Comparisons between PCI versus non-PCI groups in the overall population. **Figure S1.** Missing value in the study. **Figure S2.** Sensitivity analysis in patients with pulmonary infection. **Figure S3.** Sensitivity analysis in non-surgical patients.

Additional file 2. R code for the main analysis.

## Data Availability

Data were fully available at https://eicu-crd.mit.edu/.

## Abbreviations

AUC: Area under the curve
ICU: Intensive care unit
MOFS: Multiple organ failure syndrome
PCI: Persistent critical illness
SOFA: Sequential Organ Failure Assessment

## References

[1] Efron PA, Mohr AM, Bihorac A, Horiguchi H, Hollen MK, Segal MS (2018). Persistent inflammation, immunosuppression, and catabolism and the development of chronic critical illness after surgery. Surgery..

[2] Iwashyna TJ, Hodgson CL, Pilcher D, Bailey M, van Lint A, Chavan S (2016). Timing of onset and burden of persistent critical illness in Australia and New Zealand: a retrospective, population-based, observational study. Lancet Respir Med.

[3] Bagshaw SM, Stelfox HT, Iwashyna TJ, Bellomo R, Zuege D, Wang X (2018). Timing of onset of persistent critical illness: a multi-centre retrospective cohort studySpringer. Intensive Care Med.

[4] van Vught LA, Klein Klouwenberg PMC, Spitoni C, Scicluna BP, Wiewel MA, Horn J (2016). Incidence, risk factors, and attributable mortality of secondary infections in the intensive care unit after admission for sepsis. JAMA..

[5] Zhou F, Li H, Gu L, Liu M, Xue C-X, Cao B (2018). Risk factors for nosocomial infection among hospitalised severe influenza a(H1N1)pdm09 patients. Respir Med.

[6] Philippart F, Bouroche G, Timsit J-F, Garrouste-Orgeas M, Azoulay E, Darmon M (2015). Decreased risk of ventilator-associated pneumonia in sepsis due to intra-abdominal infection. Yende S, editor. PLoS One.

[7] Hermans G, Van den Berghe G (2015). Clinical review: intensive care unit acquired weakness. Crit Care.

[8] Witteveen Esther, Wieske Luuk, van der Poll Tom, van der Schaaf Marike, van Schaik Ivo N., Schultz Marcus J., Verhamme Camiel, Horn Janneke (2017). Increased Early Systemic Inflammation in ICU-Acquired Weakness; A Prospective Observational Cohort Study*. Critical Care Medicine.

[9] Tsuruta R, Oda Y (2016). A clinical perspective of sepsis-associated delirium. J Intensive Care.

[10] Smith HAB, Gangopadhyay M, Goben CM, Jacobowski NL, Chestnut MH, Thompson JL (2017). Delirium and benzodiazepines associated with prolonged ICU stay in critically ill infants and young children. Crit Care Med.

[11] Yamaguchi T, Tsukioka E, Kishi Y (2014). Outcomes after delirium in a Japanese intensive care unit. Gen Hosp Psychiatry.

[12] Latronico N, Herridge M, Hopkins RO, Angus D, Hart N, Hermans G (2017). The ICM research agenda on intensive care unit-acquired weakness. Intensive Care Med.

[13] Shankar-Hari M, Harrison DA, Rowan KM (2016). Differences in impact of definitional elements on mortality precludes international comparisons of sepsis epidemiology-a cohort study illustrating the need for standardized reporting. Crit Care Med.

[14] Haines RW, Zolfaghari P, Wan Y, Pearse RM, Puthucheary Z, Prowle JR (2019). Elevated urea-to-creatinine ratio provides a biochemical signature of muscle catabolism and persistent critical illness after major trauma. Intensive Care Med.

[15] Pollard TJ, Johnson AEW, Raffa JD, Celi LA, Mark RG, Badawi O (2018). The eICU collaborative research database, a freely available multi-center database for critical care research. Sci Data.

[16] Zimmerman JE, Kramer AA, McNair DS, Malila FM (2006). Acute physiology and chronic health evaluation (APACHE) IV: hospital mortality assessment for today's critically ill patients. Crit Care Med.

[17] Levy MM, Fink MP, Marshall JC, Abraham E, Angus D, Cook D, et al. SCCM/ESICM/ACCP/ATS/SIS International Sepsis Definitions Conference. Intensive Care Med. 2001;2003(4):530–8. 10.1007/s00134-003-1662-x 12664219

[18] Singer M, Deutschman CS, Seymour CW, Shankar-Hari M, Annane D, Bauer M, et al. The Third International Consensus Definitions for Sepsis and Septic Shock (Sepsis-3). JAMA. 2016;315(8):801–10. 10.1001/jama.2016.0287 PMC4968574 26903338

[19] Zhang Z (2016). Multiple imputation for time series data with Amelia package. Ann Transl Med..

[20] Doove LL, Van Buuren S, Dusseldorp E (2014). Recursive partitioning for missing data imputation in the presence of interaction effects. Comput Stat Data Anal.

[21] Zhang Z (2016). Multiple imputation with multivariate imputation by chained equation (MICE) package. Ann Transl Med..

[22] Bauer DJ, Curran PJ (2003). Distributional assumptions of growth mixture models: implications for overextraction of latent trajectory classes. Psychol Methods.

[23] Jo B, Findling RL, Wang C-P, Hastie TJ, Youngstrom EA, Arnold LE (2017). Targeted use of growth mixture modeling: a learning perspective. Stat Med.

[24] Nagin DS, Odgers CL (2010). Group-based trajectory modeling in clinical research. Annu Rev Clin Psychol.

[25] Proust-Lima C, Philipps V, Liquet B. Estimation of extended mixed models using latent classes and latent processes: the R Package lcmm. J Stat Softw. 2017;78(2):1-56.

[26] Nylund KL, Asparouhov T, Muthén BO. Deciding on the number of classes in latent class analysis and growth mixture modeling: a Monte Carlo simulation study. Struct Equ Modeling. 4 ed. Taylor & Francis Group; 2007;14:535–569.

[27] Fisher LD, Lin DY (1999). Time-dependent covariates in the cox proportional-hazards regression model. Annu Rev Public Health.

[28] Zhang Zhongheng, Reinikainen Jaakko, Adeleke Kazeem Adedayo, Pieterse Marcel E., Groothuis-Oudshoorn Catharina G. M. (2018). Time-varying covariates and coefficients in Cox regression models. Annals of Translational Medicine.

[29] Mira JC, Cuschieri J, Ozrazgat-Baslanti T, Wang Z, Ghita GL, Loftus TJ (2017). The epidemiology of chronic critical illness after severe traumatic injury at two level-one trauma centers. Crit Care Med.

[30] Iwashyna TJ, Hodgson CL, Pilcher D, Bailey M, Bellomo R (2015). Persistent critical illness characterised by Australian and New Zealand ICU clinicians. Crit Care Resusc.

[31] Moore FA, Sauaia A, Moore EE, Haenel JB, Burch JM, Lezotte DC (1996). Postinjury multiple organ failure: a bimodal phenomenon. J Trauma.

[32] Dewar DC, Tarrant SM, King KL, Balogh ZJ (2013). Changes in the epidemiology and prediction of multiple-organ failure after injury. J Trauma Acute Care Surg.

[33] Minei JP, Cuschieri J, Sperry J, Moore EE, West MA, Harbrecht BG (2012). The changing pattern and implications of multiple organ failure after blunt injury with hemorrhagic shock. Crit Care Med.

[34] Sauaia A, Moore EE, Johnson JL, Chin TL, Banerjee A, Sperry JL (2014). Temporal trends of postinjury multiple-organ failure: still resource intensive, morbid, and lethal. J Trauma Acute Care Surg.

[35] Raith EP, Udy AA, Bailey M, McGloughlin S, MacIsaac C, Bellomo R (2017). Prognostic accuracy of the SOFA score, SIRS criteria, and qSOFA score for in-hospital mortality among adults with suspected infection admitted to the intensive care unit. JAMA.

