# Supplementary material for: Defining persistent critical illness based on growth trajectories in patients with sepsis
Source: Crit Care. 2020 Feb 18;24:57. doi: 10.1186/s13054-020-2768-z (PMC7029548; doi:10.1186/s13054-020-2768-z)
Supplement: Supplementary file 1 — Table S1. Fixed effects in the longitudinal 5-class model. Table S2. Predictive performance of acute and antecedent models as represented by AUCs for the day 1, 2, 7, 14 and 21. Table S3. The differences of baseline variables across the 5 classes. Table S4. Binary logistic regression model using antecedent variables to predict mortality. Table S5. Binary logistic regression model using acute variables to predict mortality. Table S6. Comparisons between PCI versus non-PCI groups in the overall population. Figure S1. Missing value in the study. Figure S2. Sensitivity analysis in patients with pulmonary infection. Figure S3. Sensitivity analysis in non-surgical patients. [file 13054_2020_2768_MOESM1_ESM.docx]

# Latent growth mixture modeling

Latent growth mixture modeling assumes that the population is heterogeneous and composed of J latent classes of subjects characterized by J mean profiles of trajectories [1]. The outcome $Y$ (SOFA score) for individual $i$ at time point $t$ conditional on trajectory class $c_{i}$ is expressed as [2]:

$$Y_{it}|_{c_{i}=j}=\eta_{1ij}+\eta_{2ij}S_{t}+\eta_{3ij}{S_{t}}^{2}+\omega_{ijt}$$

$$\eta_{1ij}=\eta_{1j}+\zeta_{1ij}$$

$$\eta_{2ij}=\eta_{2j}+\zeta_{2ij}$$

$$\eta_{3ij}=\eta_{3j}+\zeta_{3ij}$$

Where there were J possible trajectory classes. A quadratic growth model was chosen to capture potentially nonlinear patterns across ICU days. The three main growth parameters were specified as the initial status $\eta_{1j}$, linear growth $\eta_{2j}$ and the quadratic growth $\eta_{3j}$ for trajectory j. The time score (ICU days) $S_{t}$ is the linear and ${S_{t}}^{2}$ the quadratic growth[3,4]. No covariates were included to predict class membership. The best number of classes was determined by statistics such as Akaike information criterion (AIC), Bayesian information criteria (BIC), sample-adjusted BIC and entropy. A smaller AIC, BIC SABIC and entropy value indicated a better model fit [5]. Because a substantial number of patients are required for each class to be robust and clinically meaningful, 500 subjects was predefined as the minimum sample size required for each class. The R package *lcmm* (version 1.7.9) was used for the Latent growth mixture modeling.

Cox hazard model with time-dependent coefficient was employed to further test the hypothesis that the predictive performance of acute variables would attenuate with time. The conventional Cox proportional hazard model was extended by allowing the coefficient to vary over time [6,7]:

$$\lambda\left( t \right)=\lambda_{0}\left( t \right)e^{\beta\left( t \right)X}$$

where $\lambda\left( t \right)$ is the hazard function and $\lambda_{0}\left( t \right)$ is the baseline hazard function. $\beta\left( t \right)$ is the coefficient varies over time. For the ease of interpretation, we specified a step function for $\beta\left( t \right)$, i.e. different coefficients over different time intervals (0 - 48 hours, 48 -72 hours, 72 hours - 7 days, 7 – 14 days, 14 – 21 days and >21 days). Two logistic regression models were built by regressing mortality on acute and antecedent variables, respectively. Acute variables were aggregated into an acute score reflecting the propensity to have the event conditional on these acute variables. Similarly, an antecedent score was calculated for each subject. Then both acute and antecedent scores were entered into the Cox hazard model with time-dependent coefficient.

Table S1 Fixed effects in the longitudinal 5-class model

|  | Coefficient | Standard error | Wald statistic | p-value |
| --- | --- | --- | --- | --- |
| Intercept class1 | 10.13421 | 0.14631 | 69.265 | < 0.001 |
| Intercept class2 | 13.70936 | 0.11707 | 117.106 | < 0.001 |
| Intercept class3 | 6.24705 | 0.04137 | 151.021 | < 0.001 |
| Intercept class4 | 7.15874 | 0.13608 | 52.607 | < 0.001 |
| Intercept class5 | 11.31168 | 0.10666 | 106.058 | < 0.001 |
| poly1 class1 | -0.52516 | 0.04424 | -11.872 | < 0.001 |
| poly1 class2 | 0.23245 | 0.05107 | 4.552 | 0.00001 |
| poly1 class3 | -0.12643 | 0.02224 | -5.685 | < 0.001 |
| poly1 class4 | 0.51318 | 0.06584 | 7.794 | < 0.001 |
| poly1 class5 | 0.08924 | 0.03802 | 2.347 | 0.01892 |
| poly2 class1 | 0.02084 | 0.00355 | 5.873 | < 0.001 |
| poly2 class2 | -0.03294 | 0.00483 | -6.823 | < 0.001 |
| poly2 class3 | 0.01724 | 0.00249 | 6.924 | < 0.001 |
| poly2 class4 | -0.02638 | 0.00544 | -4.853 | < 0.001 |
| poly2 class5 | -0.02674 | 0.00333 | -8.037 | < 0.001 |

Note: Polynomials up to a power of two was allowed for the ICU days. Poly1 indicates the first order (linear) term and the poly2 indicates the second order (quadratic) term. The full model comprises an intercept term, first and second order terms for each class. Note there are 5 classes in the longitudinal model. Random-effects are also allowed for the model.

Table S2. Predictive performance of acute and antecedent models as represented by AUCs for the day 1, 2, 7, 14 and 21

|  | Models | Day 1 | Day 2 | Day 7 | Day 14 | Day 21 |
| --- | --- | --- | --- | --- | --- | --- |
| Overall population | Acute | 0.764(0.749,0.776) | 0.738(0.727,0.755) | 0.661(0.633,0.677) | 0.595(0.553,0.643) | 0.596(0.537,0.654) |
|  | Antecedent | 0.619(0.604,0.634) | 0.615(0.596,0.63) | 0.581(0.558,0.608) | 0.587(0.556,0.63) | 0.585(0.525,0.639) |
| Class 1 | Acute | 0.681(0.655,0.715) | 0.659(0.616,0.694) | 0.613(0.548,0.671) | 0.602(0.475,0.665) | 0.57(0.446,0.699) |
|  | Antecedent | 0.624(0.589,0.662) | 0.619(0.58,0.649) | 0.582(0.531,0.629) | 0.558(0.477,0.634) | 0.596(0.478,0.761) |
| Class 2 | acute | 0.655(0.589,0.717) | 0.616(0.549,0.689) | 0.621(0.508,0.704) | 0.668(0.525,0.785) | 0.689(0.469,0.87) |
|  | Antecedent | 0.601(0.527,0.654) | 0.601(0.553,0.671) | 0.584(0.495,0.676) | 0.635(0.499,0.735) | 0.572(0.466,0.713) |
| Class 3 | acute | 0.702(0.664,0.73) | 0.68(0.648,0.71) | 0.6(0.543,0.653) | 0.528(0.478,0.589) | 0.554(0.462,0.684) |
|  | Antecedent | 0.654(0.628,0.677) | 0.65(0.615,0.681) | 0.584(0.527,0.632) | 0.533(0.46,0.617) | 0.559(0.456,0.665) |
| Class 4 | acute | 0.665(0.632,0.7) | 0.659(0.613,0.695) | 0.589(0.529,0.642) | 0.532(0.464,0.602) | 0.562(0.425,0.679) |
|  | Antecedent | 0.633(0.595,0.667) | 0.628(0.59,0.669) | 0.591(0.535,0.629) | 0.608(0.489,0.708) | 0.609(0.485,0.746) |
| Class 5 | acute | 0.665(0.63,0.7) | 0.654(0.618,0.702) | 0.625(0.57,0.67) | 0.538(0.476,0.613) | 0.591(0.461,0.682) |
|  | Antecedent | 0.621(0.582,0.649) | 0.606(0.563,0.636) | 0.569(0.488,0.625) | 0.610(0.537,0.698) | 0.582(0.441,0.723) |

Note: the discriminations of the antecedent and acute models are represented by the area under the curve (AUC). Each model was trained in 70% of the whole dataset, and then validated in the remaining 30% patients.

Table S3. The differences of baseline variables across the 5 classes.

| **Variables** | Class 1 (n = 5211) | Class 2 (n = 812) | Class 3 (n = 11816) | Class 4 (n = 2560) | Class 5 (n = 2469) | p |
| --- | --- | --- | --- | --- | --- | --- |
| Gender, Male (%) | 2793 ( 53.6) | 465 ( 57.3) | 5623 ( 47.6) | 1346 ( 52.6) | 1373 ( 55.6) | <0.001 |
| Age (mean (SD)) | 65.97 (17.44) | 60.52 (16.19) | 64.53 (18.51) | 65.82 (17.82) | 64.27 (16.97) | <0.001 |
| Ethnicity (%) |  |  |  |  |  | <0.001 |
| African American | 550 ( 10.6) | 114 ( 14.0) | 1016 ( 8.6) | 268 ( 10.5) | 334 ( 13.5) |  |
| Asian | 101 ( 1.9) | 11 ( 1.4) | 237 ( 2.0) | 46 ( 1.8) | 43 ( 1.7) |  |
| Caucasian | 4001 ( 76.8) | 573 ( 70.6) | 9358 ( 79.2) | 1995 ( 77.9) | 1787 ( 72.4) |  |
| Hispanic | 218 ( 4.2) | 43 ( 5.3) | 438 ( 3.7) | 94 ( 3.7) | 127 ( 5.1) |  |
| Native American | 44 ( 0.8) | 14 ( 1.7) | 101 ( 0.9) | 15 ( 0.6) | 27 ( 1.1) |  |
| Other/Unknown | 297 ( 5.7) | 57 ( 7.0) | 666 ( 5.6) | 142 ( 5.5) | 151 ( 6.1) |  |
| Admission height (cm) (mean (SD)) | 168.33 (15.13) | 170.15 (11.69) | 167.86 (14.72) | 168.22 (13.46) | 168.83 (14.19) | <0.001 |
| Admission weight (kg) (mean (SD)) | 83.19 (27.34) | 87.04 (29.17) | 81.36 (28.46) | 82.00 (28.46) | 84.21 (29.35) | <0.001 |
| Source of infection (%) |  |  |  |  |  | <0.001 |
| GI | 633 ( 12.1) | 147 ( 18.1) | 1427 ( 12.1) | 283 ( 11.1) | 353 ( 14.3) |  |
| cutaneous/soft tissue | 398 ( 7.6) | 44 ( 5.4) | 1066 ( 9.0) | 203 ( 7.9) | 192 ( 7.8) |  |
| gynecologic | 14 ( 0.3) | 1 ( 0.1) | 54 ( 0.5) | 1 ( 0.0) | 5 ( 0.2) |  |
| other | 364 ( 7.0) | 87 ( 10.7) | 709 ( 6.0) | 157 ( 6.1) | 183 ( 7.4) |  |
| pulmonary | 1993 ( 38.2) | 295 ( 36.3) | 4436 ( 37.5) | 1055 ( 41.2) | 972 ( 39.4) |  |
| renal/UTI (including bladder) | 1172 ( 22.5) | 98 ( 12.1) | 2941 ( 24.9) | 569 ( 22.2) | 432 ( 17.5) |  |
| unknown | 637 ( 12.2) | 140 ( 17.2) | 1183 ( 10.0) | 292 ( 11.4) | 332 ( 13.4) |  |
| Admitting source (%) |  |  |  |  |  | <0.001 |
| Operating room | 19 ( 0.4) | 0 ( 0.0) | 26 ( 0.2) | 1 ( 0.0) | 6 ( 0.2) |  |
| Recovery Room | 10 ( 0.2) | 0 ( 0.0) | 26 ( 0.2) | 4 ( 0.2) | 1 ( 0.0) |  |
| Chest Pain Center | 1 ( 0.0) | 0 ( 0.0) | 2 ( 0.0) | 0 ( 0.0) | 0 ( 0.0) |  |
| Floor | 1187 ( 22.8) | 218 ( 26.8) | 2278 ( 19.3) | 597 ( 23.3) | 664 ( 26.9) |  |
| Other ICU | 50 ( 1.0) | 9 ( 1.1) | 70 ( 0.6) | 18 ( 0.7) | 26 ( 1.1) |  |
| Other Hospital | 112 ( 2.1) | 38 ( 4.7) | 190 ( 1.6) | 60 ( 2.3) | 89 ( 3.6) |  |
| Direct Admit | 318 ( 6.1) | 79 ( 9.7) | 623 ( 5.3) | 149 ( 5.8) | 174 ( 7.0) |  |
| Emergency Department | 3514 ( 67.4) | 468 ( 57.6) | 8601 ( 72.8) | 1731 ( 67.6) | 1509 ( 61.1) |  |
| Unit type (%) |  |  |  |  |  | <0.001 |
| CCU-CTICU | 323 ( 6.2) | 46 ( 5.7) | 689 ( 5.8) | 162 ( 6.3) | 119 ( 4.8) |  |
| CSICU | 73 ( 1.4) | 10 ( 1.2) | 204 ( 1.7) | 37 ( 1.4) | 55 ( 2.2) |  |
| CTICU | 34 ( 0.7) | 9 ( 1.1) | 82 ( 0.7) | 15 ( 0.6) | 22 ( 0.9) |  |
| Cardiac ICU | 331 ( 6.4) | 49 ( 6.0) | 663 ( 5.6) | 157 ( 6.1) | 147 ( 6.0) |  |
| MICU | 767 ( 14.7) | 118 ( 14.5) | 1384 ( 11.7) | 375 ( 14.6) | 424 ( 17.2) |  |
| Med-Surg ICU | 3366 ( 64.6) | 520 ( 64.0) | 8171 ( 69.2) | 1662 ( 64.9) | 1551 ( 62.8) |  |
| Neuro ICU | 112 ( 2.1) | 22 ( 2.7) | 195 ( 1.7) | 54 ( 2.1) | 50 ( 2.0) |  |
| SICU | 205 ( 3.9) | 38 ( 4.7) | 428 ( 3.6) | 98 ( 3.8) | 101 ( 4.1) |  |
| Use of mechanical ventilation (%) | 1763 ( 33.8) | 601 ( 74.0) | 1187 ( 10.0) | 582 ( 22.7) | 1315 ( 53.3) | <0.001 |
| GCS (median [IQR]) | 11.00 [8.00, 14.00] | 7.00 [3.00, 9.00] | 14.00 [12.41, 15.00] | 13.00 [10.06, 14.83] | 9.00 [6.06, 12.24] | <0.001 |
| Bilirubin (mg/dl) (mean (SD)) | 2.93 (2.74) | 4.98 (5.58) | 1.76 (1.92) | 1.84 (2.14) | 3.26 (3.57) | <0.001 |
| Creatinine (mg/dl) (mean (SD)) | 2.56 (2.11) | 3.45 (2.21) | 1.36 (1.08) | 1.90 (1.74) | 3.24 (2.39) | <0.001 |
| Platelet ($\times{10}^{9}/L$) (mean (SD)) | 168.79 (103.57) | 87.23 (73.40) | 220.67 (105.42) | 198.72 (106.09) | 139.38 (98.30) | <0.001 |
| PaO2 (mmHg) (mean (SD)) | 88.57 (48.16) | 80.83 (47.68) | 94.58 (45.58) | 97.12 (51.70) | 89.22 (51.38) | <0.001 |
| Mean blood pressure (mmHg) (mean (SD)) | 55.63 (16.11) | 45.81 (17.60) | 61.19 (15.34) | 58.32 (15.43) | 50.54 (16.95) | <0.001 |
| SOFA (median [IQR]) | 10.00 [9.00, 11.00] | 15.00 [13.00, 16.00] | 6.00 [5.00, 7.00] | 7.00 [6.00, 8.00] | 12.00 [10.00, 13.00] | <0.001 |
| Dialysis (%) | 445 ( 8.5) | 70 ( 8.6) | 202 ( 1.7) | 151 ( 5.9) | 329 ( 13.3) | <0.001 |
| Urine output (ml/24 hrs) (median [IQR]) | 992.13 [391.82, 1945.90] | 442.02 [83.59, 1114.65] | 1431.00 [734.05, 2519.58] | 1197.24 [557.93, 2212.64] | 649.90 [174.18, 1461.89] | <0.001 |
| WBC ($\times{10}^{9}/L$) (median [IQR]) | 13.30 [7.90, 19.87] | 12.00 [4.50, 21.63] | 13.30 [8.72, 19.00] | 13.30 [8.30, 19.70] | 13.49 [6.90, 21.50] | <0.001 |
| Temperature (℃) (median [IQR]) | 36.50 [36.10, 36.90] | 36.40 [35.70, 36.80] | 36.60 [36.30, 36.90] | 36.50 [36.20, 36.80] | 36.40 [35.90, 36.90] | <0.001 |
| Respiratory rate (/min) (median [IQR]) | 31.00 [16.00, 39.00] | 35.00 [28.00, 41.00] | 31.00 [15.00, 38.00] | 32.00 [19.50, 39.00] | 33.00 [24.00, 40.00] | <0.001 |
| Sodium (mmol/l) (mean (SD)) | 138.35 (7.18) | 137.84 (8.15) | 137.84 (5.83) | 138.21 (6.97) | 138.09 (7.37) | <0.001 |
| Heart rate (/min) (median [IQR]) | 113.00 [97.00, 130.00] | 126.00 [107.00, 141.25] | 112.00 [98.00, 126.00] | 114.00 [99.00, 130.00] | 118.00 [101.00, 135.00] | <0.001 |
| pH (mean (SD)) | 7.34 (0.12) | 7.27 (0.14) | 7.37 (0.09) | 7.36 (0.10) | 7.31 (0.13) | <0.001 |
| Hematocrit (%) (mean (SD)) | 30.38 (6.55) | 28.80 (7.51) | 31.51 (5.98) | 30.66 (6.20) | 29.78 (6.73) | <0.001 |
| Albumin (mg/dl) (mean (SD)) | 2.44 (0.61) | 2.23 (0.62) | 2.56 (0.60) | 2.43 (0.59) | 2.36 (0.64) | <0.001 |
| PaCO2 (mmHg) (mean (SD)) | 40.01 (13.32) | 37.77 (12.38) | 40.03 (13.37) | 39.33 (13.20) | 39.48 (13.59) | <0.001 |
| BUN (mean (SD)) | 42.87 (28.50) | 52.68 (31.00) | 27.45 (20.05) | 35.38 (24.93) | 50.48 (29.82) | <0.001 |
| Glucose (mean (SD)) | 168.23 (117.21) | 167.68 (131.76) | 157.64 (91.58) | 162.46 (105.53) | 170.96 (117.38) | <0.001 |
| AIDS (%) | 17 ( 0.3) | 6 ( 0.7) | 33 ( 0.3) | 6 ( 0.2) | 10 ( 0.4) | 0.177 |
| Hepatic failure (%) | 143 ( 2.7) | 76 ( 9.4) | 119 ( 1.0) | 49 ( 1.9) | 109 ( 4.4) | <0.001 |
| Lymphoma (%) | 61 ( 1.2) | 16 ( 2.0) | 93 ( 0.8) | 23 ( 0.9) | 34 ( 1.4) | 0.001 |
| Metastatic cancer (%) | 152 ( 2.9) | 35 ( 4.3) | 420 ( 3.6) | 94 ( 3.7) | 101 ( 4.1) | 0.046 |
| Leukemia (%) | 96 ( 1.8) | 38 ( 4.7) | 127 ( 1.1) | 41 ( 1.6) | 55 ( 2.2) | <0.001 |
| Immunosuppression (%) | 299 ( 5.7) | 72 ( 8.9) | 608 ( 5.1) | 141 ( 5.5) | 179 ( 7.2) | <0.001 |
| Cirrhosis (%) | 197 ( 3.8) | 116 ( 14.3) | 145 ( 1.2) | 64 ( 2.5) | 119 ( 4.8) | <0.001 |

Abbreviations: GI: gastrointestinal; UTI: urinary tract infection; ICU: intensive care unit; CCU: coronary care unit; CTICU: Cardiothoracic Intensive Care Unit; CSICU: Cardiac Surgical Intensive Care; MICU: medical ICU; SICU: surgical ICU; GCS: Glasgow coma scale; SOFA: sequential organ failure assessment; WBC: white blood cell count; BUN: blood urea nitrogen; AIDS: acquired immunodeficiency syndrome; SD: standard deviation; IQR: interquartile range; SMD: standardized mean difference.

Table S4. Binary logistic regression model using antecedent variables to predict mortality

| Variables | OR (95% CI) | p |
| --- | --- | --- |
| (Intercept) | 0.04 [0.03, 0.05] | <0.001 |
| Age | 1.02 [1.02, 1.03] | <0.001 |
| Gender (female as reference) | 0.99 [0.92, 1.06] | 0.825 |
| AIDS | 2.58 [1.45, 4.40] | 0.001 |
| Hepatic failure | 1.56 [1.22, 1.98] | <0.001 |
| Lymphoma | 1.31 [0.95, 1.79] | 0.095 |
| Metastatic cancer | 1.97 [1.66, 2.33] | <0.001 |
| Leukemia | 1.86 [1.46, 2.37] | <0.001 |
| immunosuppression | 1.28 [1.10, 1.48] | 0.001 |
| Cirrhosis | 2.34 [1.88, 2.88] | <0.001 |

Abbreviations: AIDS: acquired immunodeficiency syndrome.

Table S5. Binary logistic regression model using acute variables to predict mortality

| Variables | OR (95% CI) | p |
| --- | --- | --- |
| (Intercept) | 163.51 [39.86, 676.16] | <0.001 |
| GCS | 0.95 [0.94, 0.97] | <0.001 |
| Bilirubin | 1.01 [0.99, 1.02] | 0.409 |
| Creatinine | 0.91 [0.89, 0.94] | <0.001 |
| Platelet | 1.00 [1.00, 1.00] | 0.021 |
| PaO2 | 1.00 [1.00, 1.00] | 0.098 |
| FiO2 | 1.00 [1.00, 1.01] | <0.001 |
| Mean blood pressure | 0.98 [0.98, 0.98] | <0.001 |
| SOFA | 1.17 [1.14, 1.21] | <0.001 |
| PEEP | 1.08 [0.97, 1.20] | 0.184 |
| WBC | 1.01 [1.01, 1.01] | <0.001 |
| Temperature | 0.85 [0.83, 0.88] | <0.001 |
| Respiratory rate | 1.02 [1.01, 1.02] | <0.001 |
| Sodium | 0.99 [0.99, 1.00] | 0.064 |
| Heart rate | 1.00 [1.00, 1.01] | <0.001 |
| Hematocrit | 1.00 [1.00, 1.01] | 0.223 |
| Albumin | 0.58 [0.54, 0.62] | <0.001 |
| BUN | 1.01 [1.01, 1.01] | <0.001 |
| Glucose | 1.00 [1.00, 1.00] | <0.001 |

OR was reported for each one unit increase in each of the variables.

Multicollinearity was examined using variance inflation factor and all continuous variables showed a VIF less than 5, indicating no significant multicollinearity.

Abbreviations: GCS: Glasgow coma scale; SOFA: sequential organ failure assessment; PEEP: positive end expiratory pressure; WBC: white blood cell count; BUN: blood urea nitrogen.

Table S6 Comparisons between PCI versus non-PCI groups in the overall population

| Variables | Total (n = 22868) | Non-PCI (n = 22225) | PCI (n = 643) | p |
| --- | --- | --- | --- | --- |
| Age, Mean ± SD | 64.83 ± 17.99 | 65.02 ± 17.98 | 58.47 ± 17.03 | < 0.001 |
| Gender, n (%) |  |  |  | 0.046 |
|  | 1 (0) | 1 (0) | 0 (0) |  |
| Female | 11265 (49) | 10978 (49) | 287 (45) |  |
| Male | 11600 (51) | 11244 (51) | 356 (55) |  |
| Ethnicity, n (%) |  |  |  | 0.036 |
|  | 200 (1) | 196 (1) | 4 (1) |  |
| African American | 2282 (10) | 2203 (10) | 79 (12) |  |
| Asian | 438 (2) | 421 (2) | 17 (3) |  |
| Caucasian | 17714 (77) | 17239 (78) | 475 (74) |  |
| Hispanic | 920 (4) | 883 (4) | 37 (6) |  |
| Native American | 201 (1) | 198 (1) | 3 (0) |  |
| Other/Unknown | 1113 (5) | 1085 (5) | 28 (4) |  |
| Site of infection, n (%) |  |  |  | < 0.001 |
| Sepsis, GI | 2843 (12) | 2747 (12) | 96 (15) |  |
| Sepsis, cutaneous/soft tissue | 1903 (8) | 1841 (8) | 62 (10) |  |
| Sepsis, gynecologic | 75 (0) | 74 (0) | 1 (0) |  |
| Sepsis, other | 1500 (7) | 1470 (7) | 30 (5) |  |
| Sepsis, pulmonary | 8751 (38) | 8411 (38) | 340 (53) |  |
| Sepsis, renal/UTI (including bladder) | 5212 (23) | 5144 (23) | 68 (11) |  |
| Sepsis, unknown | 2584 (11) | 2538 (11) | 46 (7) |  |
| Unit type, n (%) |  |  |  | < 0.001 |
| CCU-CTICU | 1339 (6) | 1291 (6) | 48 (7) |  |
| CSICU | 379 (2) | 368 (2) | 11 (2) |  |
| CTICU | 162 (1) | 154 (1) | 8 (1) |  |
| Cardiac ICU | 1347 (6) | 1286 (6) | 61 (9) |  |
| MICU | 3068 (13) | 2982 (13) | 86 (13) |  |
| Med-Surg ICU | 15270 (67) | 14896 (67) | 374 (58) |  |
| Neuro ICU | 433 (2) | 416 (2) | 17 (3) |  |
| SICU | 870 (4) | 832 (4) | 38 (6) |  |
| Admission height, Mean ± SD | 168.2 ± 14.54 | 168.16 ± 14.6 | 169.64 ± 12.24 | 0.003 |
| GCS, Median (IQR) | 13 (10, 15) | 13.14 (10, 15) | 9 (6.18, 12.18) | < 0.001 |
| SOFA, Median (IQR) | 8 (6, 10) | 8 (6, 10) | 10 (8, 12) | < 0.001 |
| Hospital discharge location, n (%) |  |  |  | < 0.001 |
|  | 30 (0) | 27 (0) | 3 (0) |  |
| Death | 3828 (17) | 3665 (17) | 163 (26) |  |
| Home | 9368 (43) | 9281 (43) | 87 (14) |  |
| Nursing Home | 414 (2) | 387 (2) | 27 (4) |  |
| Other External | 1548 (7) | 1489 (7) | 59 (9) |  |
| Other Hospital | 1127 (5) | 1041 (5) | 86 (14) |  |
| Rehabilitation | 778 (4) | 714 (3) | 64 (10) |  |
| Skilled Nursing Facility | 4866 (22) | 4732 (22) | 134 (22) |  |
| Unit discharge status, n (%) |  |  |  | < 0.001 |
|  | 4 (0) | 4 (0) | 0 (0) |  |
| Alive | 20448 (89) | 19914 (90) | 534 (83) |  |
| Expired | 2416 (11) | 2307 (10) | 109 (17) |  |

Figure S1 Missing value in the study.

Variables such as urine output, PaCO2 and pH showed missing rates over 50% and were excluded from the logistic regression models.

Figure S2. Sensitivity analysis in patients with pulmonary infection.

The figure shows that the transition time for septic patients with pulmonary infection is 13 days.

Figure S3. Sensitivity analysis in non-surgical patients.

The figure shows that the transition time for non-surgical patients with pulmonary infection is 20 days.

# Reference

1. Bauer DJ, Curran PJ. Distributional assumptions of growth mixture models: implications for overextraction of latent trajectory classes. Psychological Methods. 2003;8:338–63.

2. Jo B, Findling RL, Wang C-P, Hastie TJ, Youngstrom EA, Arnold LE, et al. Targeted use of growth mixture modeling: a learning perspective. Stat Med. 2017;36:671–86.

3. Nagin DS, Odgers CL. Group-based trajectory modeling in clinical research. Annu Rev Clin Psychol. 2010;6:109–38.

4. Proust-Lima C, Philipps V, Liquet B. Estimation of Extended Mixed Models Using Latent Classes and Latent Processes: The RPackage lcmm. Journal of Statistical Software. 2017;78.

5. Nylund KL, Asparouhov T, Muthén BO. Deciding on the Number of Classes in Latent Class Analysis and Growth Mixture Modeling: A Monte Carlo Simulation Study. Struct Equ Modeling. 4 ed. Taylor & Francis Group; 2007;14:535–69.

6. Fisher LD, Lin DY. TIME-DEPENDENT COVARIATES IN THE COX PROPORTIONAL-HAZARDS REGRESSION MODEL. Annual Review of Public Health. 1999;20:145–57.

7. Zhang Z, Reinikainen J, Adeleke KA, Pieterse ME, Groothuis-Oudshoorn CGM. Time-varying covariates and coefficients in Cox regression models. Ann Transl Med. 2018;6:121–1.
