# Supplementary material for: Defining persistent critical illness based on growth trajectories in patients with sepsis
Source: Crit Care. 2020 Feb 18;24:57. doi: 10.1186/s13054-020-2768-z (PMC7029548; doi:10.1186/s13054-020-2768-z)
Supplement: Supplementary file 2 — R code for the main analysis. [file 13054_2020_2768_MOESM2_ESM.docx]

# #latent growth mixture modeling

library(lcmm);#this package proceed very slowly and cannot get results if there are hundreds of time points (346).

m1 <- hlme(SOFA ~ poly(ICUdays, degree = 2, raw = TRUE),

subject = 'patientunitstayid', ng = 1,

data = dtcom)

m2 <- gridsearch(rep = 5, maxiter = 10, minit = m1,

hlme(SOFA ~ poly(ICUdays, degree = 2, raw = TRUE),

mixture = ~ poly(ICUdays, degree = 2, raw = TRUE),

subject = 'patientunitstayid', ng = 2,

data = dtcom))

m3 <- gridsearch(rep = 5, maxiter = 10, minit = m1,

hlme(SOFA ~ poly(ICUdays, degree = 2, raw = TRUE),

mixture = ~ poly(ICUdays, degree = 2, raw = TRUE),

subject = 'patientunitstayid', ng = 3,

data = dtcom))

m4 <- gridsearch(rep = 5, maxiter = 10, minit = m1,

hlme(SOFA ~ poly(ICUdays, degree = 2, raw = TRUE),

mixture = ~ poly(ICUdays, degree = 2, raw = TRUE),

subject = 'patientunitstayid', ng = 4,

data = dtcom))

m5 <- gridsearch(rep = 5, maxiter = 10, minit = m1,

hlme(SOFA ~ poly(ICUdays, degree = 2, raw = TRUE),

mixture = ~ poly(ICUdays, degree = 2, raw = TRUE),

subject = 'patientunitstayid', ng = 5,

data = dtcom))

m6 <- hlme(SOFA ~ poly(ICUdays, degree = 2, raw = TRUE),

mixture = ~ poly(ICUdays, degree = 2, raw = TRUE),

subject = 'patientunitstayid', ng = 6,

data = dtcom)

summarytable(m1,m2,m3,m4,m5,m6,

which = c("G", "loglik", "conv", "npm", "AIC",

"BIC", "SABIC", "entropy", "%class"))

plot(m5, which = "fit", var.time = "ICUdays",

bty = "l", ylab = "Mean SOFA",

xlab = "ICU days", lwd = 2,

marg = T,legend=NULL,shades = T)

text(2,6,paste(colnames(postprob(m5)[[1]])[1],' (',round(postprob(m5)[[1]][2,1],1),'%',')',sep = ''))

text(2,7.5,paste(colnames(postprob(m5)[[1]])[2],' (',round(postprob(m5)[[1]][2,2],1),'%',')',sep = ''))

text(2,9,paste(colnames(postprob(m5)[[1]])[3],' (',round(postprob(m5)[[1]][2,3],1),'%',')',sep = ''))

text(2,11,paste(colnames(postprob(m5)[[1]])[4],' (',round(postprob(m5)[[1]][2,4],1),'%',')',sep = ''))

text(2,14,paste(colnames(postprob(m5)[[1]])[5],' (',round(postprob(m5)[[1]][2,5],1),'%',')',sep = ''))

dtclass <- m5$pprob[,1:2]

#predictive value of acute physiological versus chronic values

apacheApsVar <- fread(input="zcat < /Users/zhang/Documents/eicu/apacheApsVar.csv.gz",

sep = ',',header = T)

apacheApsVar <- apacheApsVar[,lapply(.SD, function(xx) ifelse(xx==-1,NA,xx))]

apachePredVar <- fread(input="zcat < /Users/zhang/Documents/eicu/apachePredVar.csv.gz",

sep = ',',header = T)

apachePredVar <- apachePredVar[,lapply(.SD, function(xx) ifelse(xx==-1,NA,xx))]

dt1day <- merge(dt1day,apacheApsVar[,c(2:8,10:18,20,22:24)],

by='patientunitstayid',

all.x = T,all.y = F)

dt1day <- merge(dt1day,apachePredVar[,c(2,11,21:27)],

by='patientunitstayid',

all.x = T,all.y = F)

#missing value exploration

library(DataExplorer)

plot_missing(dt1day)

#missing value

library(mice)

imp <- mice(dt1day,m=1,seed=123, method = "cart")

dt1dayfull <- complete(imp,action = 1L)

dt1dayfull$apacheadmissiondx <- factor(dt1dayfull$apacheadmissiondx)

dt1dayfull$ethnicity <- as.factor(ifelse(dt1dayfull$ethnicity=="",

"Other/Unknown",as.character(dt1dayfull$ethnicity)))

dt1dayfull$gender <- as.factor(ifelse(dt1dayfull$gender=="",

"Female",as.character(dt1dayfull$gender)))

#1: Operating Room; 2: Recovery Room; 3: Chest Pain Center;

#4:Floor; 5:Other ICU; 6:Other Hospital

#7:Direct Admit; 8: Emergency Department

library(tableone)

var_tab1 <- c("gender","age","ethnicity",

"admissionheight","admissionweight","apacheadmissiondx",

"admitsource",

"unittype","PEEPflg","GCScore",

"bilirubinval","crval","plateletval",

"pao2val","mBP","SOFA",

"SOFAresp","SOFAgcs","SOFAcirc","SOFAliver","SOFAcoag","SOFArenal",

"class","dialysis",

"urine","wbc","temperature","respiratoryrate","sodium",

"heartrate","ph","hematocrit","albumin","pco2",

"bun","glucose","aids","hepaticfailure",

"lymphoma","metastaticcancer","leukemia","immunosuppression",

"cirrhosis")

tab1 <- CreateTableOne(vars = var_tab1,

factorVars=c("aids","hepaticfailure",

"lymphoma","metastaticcancer","leukemia","immunosuppression",

"cirrhosis","class","dialysis",

"admitsource"),

strata = c("hospitaldischargestatus"),

data = dt1dayfull)

print(tab1, nonnormal = c("GCScore","SOFA",

"SOFAresp","SOFAgcs","SOFAcirc","SOFAliver","SOFAcoag","SOFArenal",

"urine","wbc","temperature",

"respiratoryrate","heartrate"),

quote=T,

smd = TRUE)

#table showing characteristics across classes.

dt.acrossclass <- dt1dayfull[,c('class',"hospitaldischargelocation",

"unitdischargestatus","unitdischargeoffset",

"hospitaldischargestatus","hospitaldischargeoffset")]

dt.acrossclass$unitdischargeoffset <- dt.acrossclass$unitdischargeoffset/(60*24)

dt.acrossclass$hospitaldischargeoffset <- dt.acrossclass$hospitaldischargeoffset/(60*24)

#showing percentage of critical chronic illness in each class

#class 1: 17 (16)

#class 2: 24 (27)

#class 3: 10 (6)

#class 4: 6 (7)

#class 5: 9 (8)

#overall population

table(dt.acrossclass$unitdischargeoffset>=15)

sum(dt.acrossclass[dt.acrossclass$unitdischargeoffset>=15,'unitdischargeoffset'])/

sum(dt.acrossclass$unitdischargeoffset)

sum(dt.acrossclass[dt.acrossclass$unitdischargeoffset>=15,'hospitaldischargeoffset'])/

sum(dt.acrossclass$hospitaldischargeoffset)

table(dt.acrossclass$hospitaldischargestatus,dt.acrossclass$unitdischargeoffset>=15)

dt.acrossclass$chronicill.tag <- ifelse(dt.acrossclass$class==1&dt.acrossclass$unitdischargeoffset>=17,'Y',

ifelse(dt.acrossclass$class==2&dt.acrossclass$unitdischargeoffset>=24,'Y',

ifelse(dt.acrossclass$class==3&dt.acrossclass$unitdischargeoffset>=10,'Y',

ifelse(dt.acrossclass$class==4&dt.acrossclass$unitdischargeoffset>=6,'Y',

ifelse(dt.acrossclass$class==5&dt.acrossclass$unitdischargeoffset>=9,'Y','N')))))

var_tab2 <- c('chronicill.tag',"hospitaldischargelocation",

"unitdischargestatus","unitdischargeoffset",

"hospitaldischargestatus","hospitaldischargeoffset")

tab2 <- CreateTableOne(vars = var_tab2,

strata = c("class"),

data = dt.acrossclass)

print(tab2, nonnormal = c("unitdischargeoffset","hospitaldischargeoffset"),

quote=T)

#baseline characteristics across classes

tab3 <- CreateTableOne(vars = var_tab1,

factorVars=c("aids","hepaticfailure",

"lymphoma","metastaticcancer","leukemia","immunosuppression",

"cirrhosis","class","dialysis",

"admitsource"),

strata = c("class"),

data = dt1dayfull)

print(tab3, nonnormal = c("GCScore","SOFA","urine","wbc","temperature",

"respiratoryrate","heartrate"),

quote=T,

smd = TRUE)

#examine AUC in a certain ICU day

library(pROC)

library(lattice)

##AUC analysis in subgroups.

p.all <- NULL;

auc.all <- NULL;

for (class in 0:5) {

if(class==0){

dfsub <- dt1dayfull;

mainlab <- 'Overall population'

} else {

dfsub <- dt1dayfull[dt1dayfull$class==class,]

mainlab <- paste('Class',class,sep = ' ')

}

auc.ante.full <- NULL #initializing

auc.acute.full <- NULL

for (day in 1:28) {

if(table(dfsub[dfsub$hospitaldischargeoffset>=24*60*day,

'hospitaldischargestatus'])[2]>=20){

df <- dfsub;

df <- df[df$hospitaldischargeoffset>=day*24*60,]

auc.ante <- NULL;

auc.acute <- NULL;

for (i in 1:100) {

ind<-sample(1:nrow(df),size = nrow(df)*0.7,replace = F)

dftrain <- df[ind,];

dftest <- df[-ind,]

mod.ante <- glm(hospitaldischargestatus~age+gender+

aids+hepaticfailure+

lymphoma+metastaticcancer+

leukemia+immunosuppression+

cirrhosis,dftrain,family = 'binomial')

mod.acute <- glm(hospitaldischargestatus~GCScore+bilirubinval+crval+plateletval+

pao2val+fio2val+mBP+

SOFA+PEEPflg+wbc+temperature+

respiratoryrate+sodium+heartrate+

ph+hematocrit+

albumin+bun+glucose,dftrain,family = 'binomial')

pred.acute <- predict(mod.acute,newdata=dftest,type='response')

auc.acute <- c(auc.acute,auc(roc(dftest$hospitaldischargestatus,pred.acute)))

pred.ante <- predict(mod.ante,newdata=dftest,type='response')

auc.ante <- c(auc.ante,auc(roc(dftest$hospitaldischargestatus,pred.ante)))

}

auc.ante.full <- cbind(auc.ante.full,auc.ante)

auc.acute.full <- cbind(auc.acute.full,auc.acute)

cat('.')

}else{break}

}

dt.auc <- data.frame(rbind(auc.ante.full,auc.acute.full))

day <- ncol(auc.ante.full);

colnames(dt.auc) <- c(paste('day',1:day,sep = ''))

dt.auc$group <- rep(c('ante','acute'),each=100);

p.value <- unlist(lapply(dt.auc[,-(day+1)],

function(xx) t.test(xx~dt.auc$group)$p.value));

p.value <- c(p.value,rep(NA,28-length(p.value)))

auc.value <- ddply(dt.auc,.(group),summarise,

aucday1=paste(round(median(day1),3),"(",

round(quantile(day1,probs = 0.025),3),

",",

round(quantile(day1,probs = 0.975),3),")",

sep = ""),

aucday2=paste(round(median(day2),3),"(",

round(quantile(day2,probs = 0.025),3),

",",

round(quantile(day2,probs = 0.975),3),")",

sep = ""),

aucday7=paste(round(median(day7),3),"(",

round(quantile(day7,probs = 0.025),3),

",",

round(quantile(day7,probs = 0.975),3),")",

sep = ""),

aucday14=paste(round(median(day14),3),"(",

round(quantile(day14,probs = 0.025),3),

",",

round(quantile(day14,probs = 0.975),3),")",

sep = ""),

aucday21=paste(round(median(day21),3),"(",

round(quantile(day21,probs = 0.025),3),

",",

round(quantile(day21,probs = 0.975),3),")",

sep = ""))

auc.all <- rbind(auc.all,auc.value)

p.all <- rbind(p.all,p.value);

dt.auc.melt <- melt(dt.auc, id.vars = "group",

measure.vars = paste('day',1:day,sep = ''))

dt.auc.melt$days <- rep(1:day,each=200);

if(class==0){

plotauc0 <- xyplot(value~days,

groups = group,cex=0.2,alpha=0.3,

data = dt.auc.melt, xlab = 'Days after ICU entry',

ylab = 'AUC for mortality prediction',

xlim = c(0,29),ylim = c(0,0.9),

panel = function(x, y, ...) {

panel.superpose(x, y, ...,

panel.groups = function(x,y, col, col.symbol, ...) {

panel.xyplot(x, y, col=col.symbol, ...)

#panel.loess(x,y, col.line=col.symbol)

panel.smoother(x,y,col.level=col.symbol,

col.line = col.symbol,

level = 0.9,

alpha.se = 0.5)

}

)

},

grid = TRUE,

auto.key = list(title='Overall population',

text=c('Acute variable model',

'Antecedent variable model'),

space='top',lines=T))

} else {

plotauc <-xyplot(value~days,

groups = group,cex=0.2,alpha=0.3,

data = dt.auc.melt, xlab = 'Days after ICU entry',

ylab = 'AUC for mortality prediction',

main=mainlab, xlim = c(0,29),ylim = c(0,0.9),

panel = function(x, y, ...) {

panel.superpose(x, y, ...,

panel.groups = function(x,y, col, col.symbol, ...) {

panel.xyplot(x, y, col=col.symbol, ...)

#panel.loess(x,y, col.line=col.symbol)

panel.smoother(x,y,col.level=col.symbol,

col.line = col.symbol,

level = 0.9,

alpha.se = 0.5)

}

)

},

grid = TRUE)

assign(paste('plotauc',class,sep = ''),plotauc)

}

}

rownames(p.all) <- paste('class',0:5,sep = ' ');

library(gridExtra);

grid.arrange(plotauc0,plotauc1,plotauc2,

plotauc3,plotauc4,plotauc5)

#added during revision by time-varying coefficient in survival model

dtSurv <- dt1dayfull[dt1dayfull$hospitaldischargeoffset>0,]

dtSurv$hospitaldischargestatus <-

as.numeric(dtSurv$hospitaldischargestatus)-1

mod.ante <- glm(hospitaldischargestatus~age+gender+

aids+hepaticfailure+

lymphoma+metastaticcancer+

leukemia+immunosuppression+

cirrhosis,dtSurv,family = 'binomial')

ShowRegTable(mod.ante,quote = T)

dtSurv$predscoreAnte <- predict(mod.ante)

mod.acute <- glm(hospitaldischargestatus~GCScore+bilirubinval+crval+plateletval+

pao2val+fio2val+mBP+

SOFA+PEEPflg+wbc+temperature+

respiratoryrate+sodium+heartrate+

hematocrit+

albumin+bun+glucose,dtSurv,family = 'binomial')

ShowRegTable(mod.acute,quote = T)

dtSurv$predscoreAcute <- predict(mod.acute)

dtSurv$tgroup <- cut(dtSurv$hospitaldischargeoffset,

breaks = c(0,48*60,72*60,24*60*7,24*60*14,24*60*21,

max(dtSurv$hospitaldischargeoffset)))

ModSurv <- coxph(Surv(time = hospitaldischargeoffset,

hospitaldischargestatus==1) ~

predscoreAcute:strata(tgroup)+predscoreAnte:strata(tgroup),

data = dtSurv)

ShowRegTable(ModSurv,quote = T)

#sensitivity analysis by restricting to non-surgical patients

library(pROC)

library(latticeExtra)

dtNonSurg <- dt1dayfull[dt1dayfull$admitsource!=1&

dt1dayfull$admitsource!=2,]

dtResp <- dt1dayfull[dt1dayfull$apacheadmissiondx=="Sepsis, pulmonary",]

##the following code will be replaced by the dataframe(dtResp/dtNonSurg).

auc.ante.full <- NULL #initializing

auc.acute.full <- NULL

for (day in 1:28) {

if(table(dtResp[dtResp$hospitaldischargeoffset>=24*60*day,

'hospitaldischargestatus'])[2]>=20){

df <- dtResp;

df <- df[df$hospitaldischargeoffset>=day*24*60,]

auc.ante <- NULL;

auc.acute <- NULL;

for (i in 1:100) {

ind<-sample(1:nrow(df),size = nrow(df)*0.7,replace = F)

dftrain <- df[ind,];

dftest <- df[-ind,]

mod.ante <- glm(hospitaldischargestatus~age+gender+

aids+hepaticfailure+

lymphoma+metastaticcancer+

leukemia+immunosuppression+

cirrhosis,dftrain,family = 'binomial')

mod.acute <- glm(hospitaldischargestatus~GCScore+bilirubinval+crval+plateletval+

pao2val+fio2val+mBP+pco2+

SOFA+PEEPflg+urine+wbc+temperature+

respiratoryrate+sodium+heartrate+

ph+hematocrit+

albumin+bun+glucose,dftrain,family = 'binomial')

pred.acute <- predict(mod.acute,newdata=dftest,type='response')

auc.acute <- c(auc.acute,auc(roc(dftest$hospitaldischargestatus,pred.acute)))

pred.ante <- predict(mod.ante,newdata=dftest,type='response')

auc.ante <- c(auc.ante,auc(roc(dftest$hospitaldischargestatus,pred.ante)))

}

auc.ante.full <- cbind(auc.ante.full,auc.ante)

auc.acute.full <- cbind(auc.acute.full,auc.acute)

cat('.')

}else{break}

}

dt.auc <- data.frame(rbind(auc.ante.full,auc.acute.full))

day <- ncol(auc.ante.full);

colnames(dt.auc) <- c(paste('day',1:day,sep = ''))

dt.auc$group <- rep(c('ante','acute'),each=100);

p.value <- unlist(lapply(dt.auc[,-(day+1)],

function(xx) t.test(xx~dt.auc$group)$p.value));

p.value <- c(p.value,rep(NA,28-length(p.value)))

p.all <- rbind(p.all,p.value);

dt.auc.melt <- melt(dt.auc, id.vars = "group",

measure.vars = paste('day',1:day,sep = ''))

dt.auc.melt$days <- rep(1:day,each=200);

plotauc0 <- xyplot(value~days,

groups = group,cex=0.2,alpha=0.3,

data = dt.auc.melt, xlab = 'Days after ICU entry',

ylab = 'AUC',

panel = function(x, y, ...) {

panel.superpose(x, y, ...,

panel.groups = function(x,y, col, col.symbol, ...) {

panel.xyplot(x, y, col=col.symbol, ...)

#panel.loess(x,y, col.line=col.symbol)

panel.smoother(x,y,col.level=col.symbol,

col.line = col.symbol,

level = 0.9,

alpha.se = 0.5)

}

)

},

grid = TRUE,

auto.key = list(title='Patients with pulmonary infection',

text=c('Acute variable model',

'Antecedent variable model'),

space='top',lines=T))

#biochemical signiture (2020-01-10)

dtcom[, PCItag := (unitdischargeoffset > 15*24*60)]

library(lattice)

library(reshape)

BioSigvarlist <- c(grep("val",names(dtcom),value = T),"SOFA")

dtBioSig <- as.data.frame(dtcom)

dtBioSig <- dtBioSig[,c("patientunitstayid","ICUdays",

"PCItag",

BioSigvarlist)]

dtBioSig$Neulymph <- dtBioSig$bandsval/dtBioSig$lymphsval

dtBioSig$UreaCr <- dtBioSig$BUNval/dtBioSig$crval

dtBioSig$ICUdays <- factor(dtBioSig$ICUdays)

dtBioSig$PCI <- factor(dtBioSig$PCItag,labels = c("No","Yes"))

#data exploration

library(DataExplorer)

plot_histogram (dtBioSig[,4:17])#some variables are severely skewed

library(ggpubr)

library(ggstatsplot)

#trajectory for biochemical signiture

PNeulymph <- ggboxplot(dtBioSig[dtBioSig$Neulymph<=quantile(dtBioSig$Neulymph,0.95,na.rm = T)&!is.na(dtBioSig$Neulymph),],

x = "ICUdays", y = "Neulymph", fill = "PCI",

xlab = "",ylab = "Neutrophil:lymphocyte Ratio")+

ylim(0, 10.1) +

stat_compare_means(aes(group = PCI),label = "p.signif")

PUreaCr <- ggboxplot(dtBioSig[dtBioSig$UreaCr<=quantile(dtBioSig$UreaCr,0.95,na.rm = T)&!is.na(dtBioSig$UreaCr),],

x = "ICUdays", y = "UreaCr", fill = "PCI",

xlab = "",ylab = "Urea:Creatinine Ratio")+

stat_compare_means(aes(group = PCI),label = "p.signif")

Palbumin <- ggboxplot(dtBioSig[dtBioSig$albuminval<=quantile(dtBioSig$albuminval,0.95,na.rm = T)&!is.na(dtBioSig$albuminval),],

x = "ICUdays", y = "albuminval", fill = "PCI",

xlab = "",ylab = "Albumin (g/dL)")+guides(fill=FALSE)+

stat_compare_means(aes(group = PCI),label = "p.signif")

PCRP <- ggboxplot(dtBioSig[dtBioSig$CRPval<=quantile(dtBioSig$CRPval,0.90,na.rm = T)&!is.na(dtBioSig$CRPval),],

x = "ICUdays", y = "CRPval", fill = "PCI",

xlab = "",ylab = "CRP (mg/L)")+guides(fill=FALSE)+

stat_compare_means(aes(group = PCI),label = "p.signif")

PSOFA <- ggboxplot(dtBioSig[!is.na(dtBioSig$SOFA),],

x = "ICUdays", y = "SOFA", fill = "PCI",

xlab = "ICU days",ylab = "SOFA")+guides(fill=FALSE)+

stat_compare_means(aes(group = PCI),label = "p.signif")

PHgb <- ggboxplot(dtBioSig[dtBioSig$Hgbval<=quantile(dtBioSig$Hgbval,0.95,na.rm = T)&!is.na(dtBioSig$Hgbval),],

x = "ICUdays", y = "Hgbval", fill = "PCI",

xlab = "ICU days",ylab = "Haemoglobin (mg/dL)")+guides(fill=FALSE)+

stat_compare_means(aes(group = PCI),label = "p.signif")

combine_plots(PNeulymph,PUreaCr,Palbumin,PCRP,PHgb,PSOFA,

ncol=2)

#comparing delta value with heatmap

dtBioSig$ICUdays <- as.numeric(dtBioSig$ICUdays)

dtBioSig <- ddply(dtBioSig,.(patientunitstayid),function(xx){

xx$ICUlos <- max(xx$ICUdays)

return(xx)

})

dtDelta <- matrix(rep(0,9*9),ncol = 9)

for (ii in 1:9) {

for (jj in (ii+1):10) {

dtReduced <- dtBioSig[dtBioSig$ICUlos>=jj&dtBioSig$PCI=="Yes",]

VecDelta <- ddply(dtReduced,.(patientunitstayid),function(xx){

xx[xx$ICUdays==jj,"UreaCr"]-xx[xx$ICUdays==ii,"UreaCr"]

})

dtDelta[ii,jj-1] <- median(VecDelta[,2],na.rm = T)

}

}

colnames(dtDelta) <- paste("D",2:10,sep = "");

rownames(dtDelta) <- paste("D",1:9,sep = "")

MpUCrPCI <- pheatmap(dtDelta,cluster_cols=F,

cluster_rows = F,

color = c("green",colorRampPalette(colors = c("green", "black", "red"))

(n = length(seq(-1,8,by=0.1))-3), "red"),

breaks = seq(-1,8,by=0.1),

main = "Changes in Urea:Creatinine Ratio in PCI")

dtDelta <- matrix(rep(0,9*9),ncol = 9)

for (ii in 1:9) {

for (jj in (ii+1):10) {

dtReduced <- dtBioSig[dtBioSig$ICUlos>=jj&dtBioSig$PCI=="No",]

VecDelta <- ddply(dtReduced,.(patientunitstayid),function(xx){

xx[xx$ICUdays==jj,"UreaCr"]-xx[xx$ICUdays==ii,"UreaCr"]

})

dtDelta[ii,jj-1] <- median(VecDelta[,2],na.rm = T)

}

}

colnames(dtDelta) <- paste("D",2:10,sep = "");

rownames(dtDelta) <- paste("D",1:9,sep = "")

library(pheatmap)

MpUCrNoPCI <- pheatmap(dtDelta,cluster_cols=F,

cluster_rows = F,

color = c("green",colorRampPalette(colors = c("green", "black", "red"))

(n = length(seq(-1,8,by=0.1))-3), "red"),

breaks = seq(-1,8,by=0.1),

main = "Changes in Urea:Creatinine Ratio in non-PCI")

a <- list(MpUCrPCI[[4]])

a[[2]] <- MpUCrNoPCI[[4]]

do.call(grid.arrange,a)

#make a table changes from arbiturary day

tabDelta <- NULL

for (ii in 1:9) {

for (jj in (ii+1):10) {

dtReduced <- dtBioSig[dtBioSig$ICUlos>=jj,]

VecDelta <- ddply(dtReduced,.(patientunitstayid,PCI),function(xx){

xx[xx$ICUdays==jj,"UreaCr"]-xx[xx$ICUdays==ii,"UreaCr"]

})

names(VecDelta)[3] <- paste("day",ii,"vs",jj,sep = "")

tabDelta<-rbind(tabDelta,as.matrix(twogrps(VecDelta,gvar = "PCI",varlist = names(VecDelta)[3])[2,]))

cat(".")

}

}

print(tabDelta,quote = T)
